# Supplementary material for: Nano-palladium is a cellular catalyst for in vivo chemistry
Source: Nat Commun. 2017 Jul 12;8:15906. doi: 10.1038/ncomms15906 (PMC5510178; doi:10.1038/ncomms15906)
Supplement: Supplementary Information [file ncomms15906-s1.pdf]

Type of file: PDF
Size of file: 0 KB
Title of file for HTML: Supplementary Information
Description: Supplementary Figures, Supplementary Tables, Supplementary Methods and Supplementary References

Type of file: PDF
Size of file: 0 KB
Title of file for HTML: Peer Review File
Description:

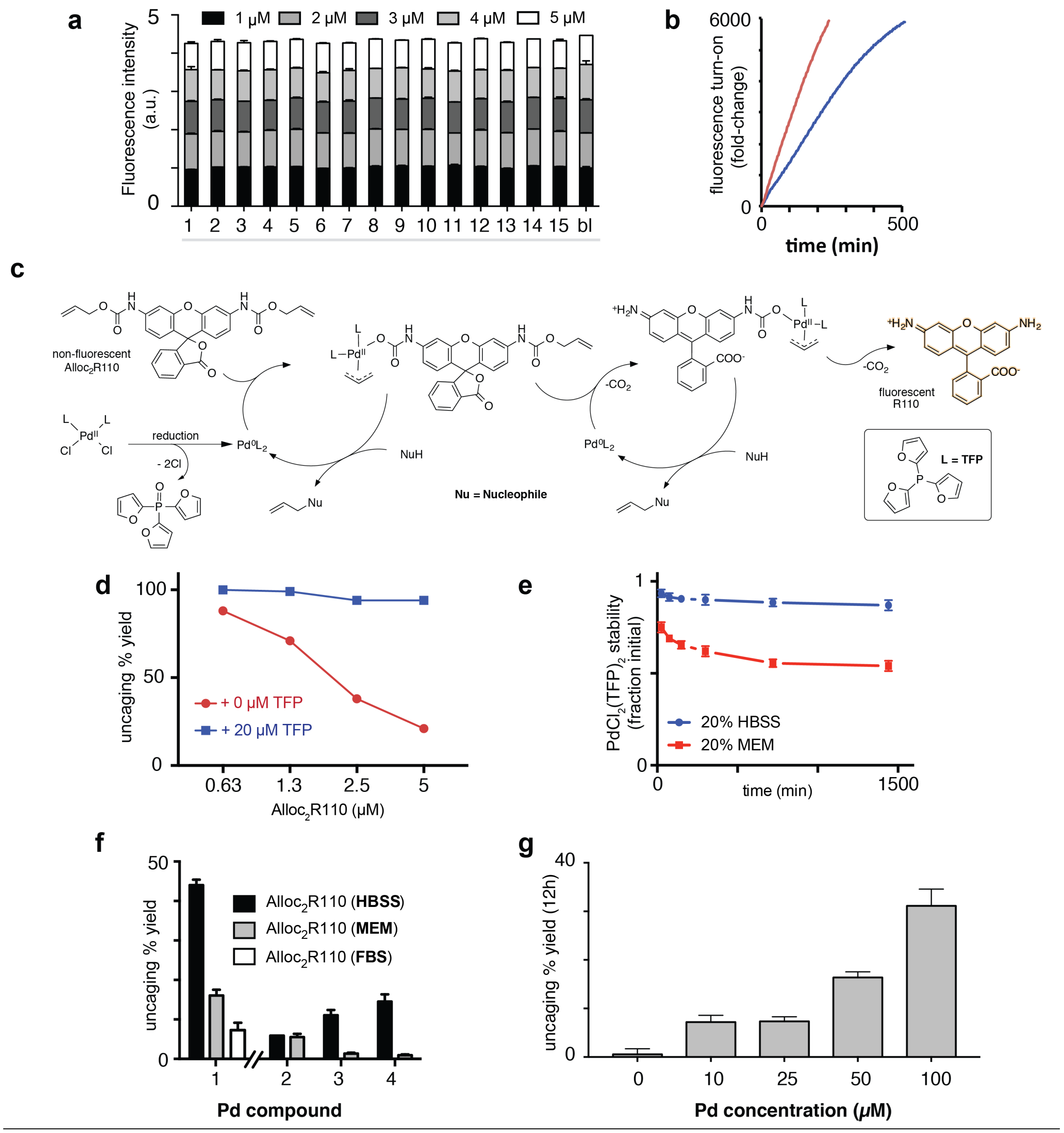


**Supplementary Figure 1. Supplemental characterization of a $PdCl_2(TFP)_2$ activity. a)** Pd compounds (10 μM) used in the catalyst screen (Fig. 1) did not cause detectable fluorescence quenching of increasing R110 concentrations in HBSS (mean ± SD, n = 2). **b)** Fluorescence turn-on of Alloc$_2$R110 (5 μM) after activation with 10 μM Pd-NP in HBSS (red) or MEM (blue) at 37°C. **c)** Scheme of $PdCl_2(TFP)_2$ reduction and catalysis of alloc$_2$R110 un-caging. **d)** TFP added to 2.5 *μ*M $PdCl_2(TFP)_2$ for 12 h at 37°C in HBSS enhances un-caging yield. **e)** $PdCl_2(TFP)_2$ stability was estimated as a ratio of $^{31}$P NMR signals from the formation of the major degradation product tri(2-furyl)phosphine oxide over time, in DMSO for solubility with either MEM or HBSS added (mean ± SD, n = 2). **f)** Alloc$_2$R110 un-caging was measured as in Fig. 1, but first with 12 h pre-incubation of catalyst in either HBSS, MEM, or FBS at 37°C (mean ± SD, n = 2). FBS was only used for compound ***1***. **g)** Alloc$_2$R110 un-caging was measured as in Fig. 1, but using 100 mg excised HT1080 xenograft tumor homogenate at 37°C and compound ***1*** at the indicated concentration (mean ± SD, n ≥ 3).

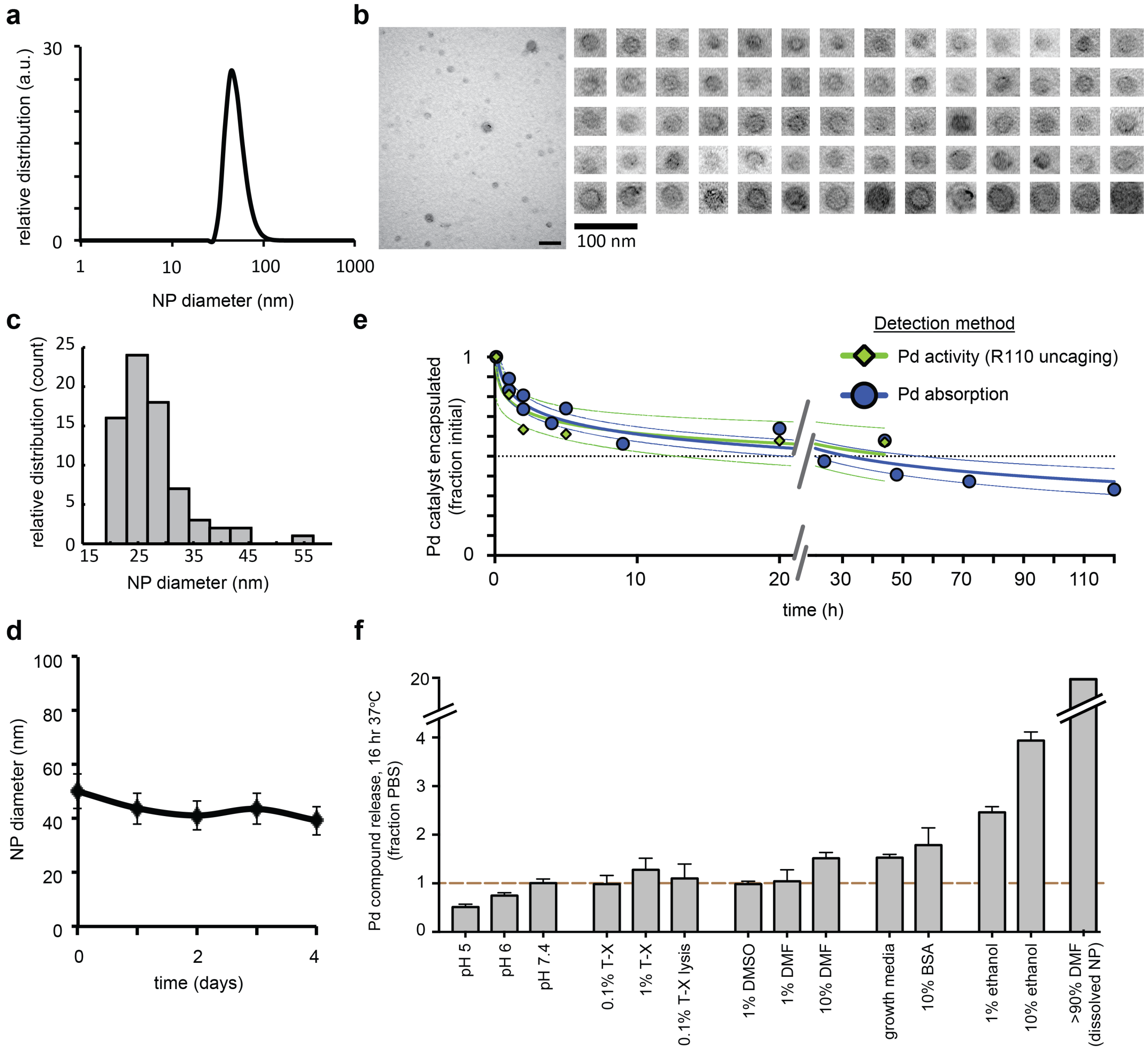

**Supplementary Figure 2. Characterization of a novel palladium nano-formulation. a)** DLS-measured distribution of Pd-NP according to diameter. **b)** TEM images of Pd-NP, corresponding to Fig. 2b, showing wider field of view (left) and individual Pd-NP compiled from multiple image replicates. **c)** Distribution of NP diameter as measured by TEM in *b*. **d)** Pd-NP is stable in HBSS at 37$^{o}$C over time (mean ± SD, n = 3), measured by DLS. **e)** *In vitro* release of $PdCl_2(TFP)_2$ from NP encapsulation, measured in PBS at 37$^{o}$C. $PdCl_2(TFP)_2$ levels were determined either using absorbance or with activation of $Alloc_2R110$ (n=2); solid and dashed lines denote semi-log fit ± 95% C.I. for each determination method. **f)** As in *e*, absorbance was used to assess release of encapsulated Pd compound in various environments, in PBS unless otherwise stated (mean ± SEM, n ≥ 2).

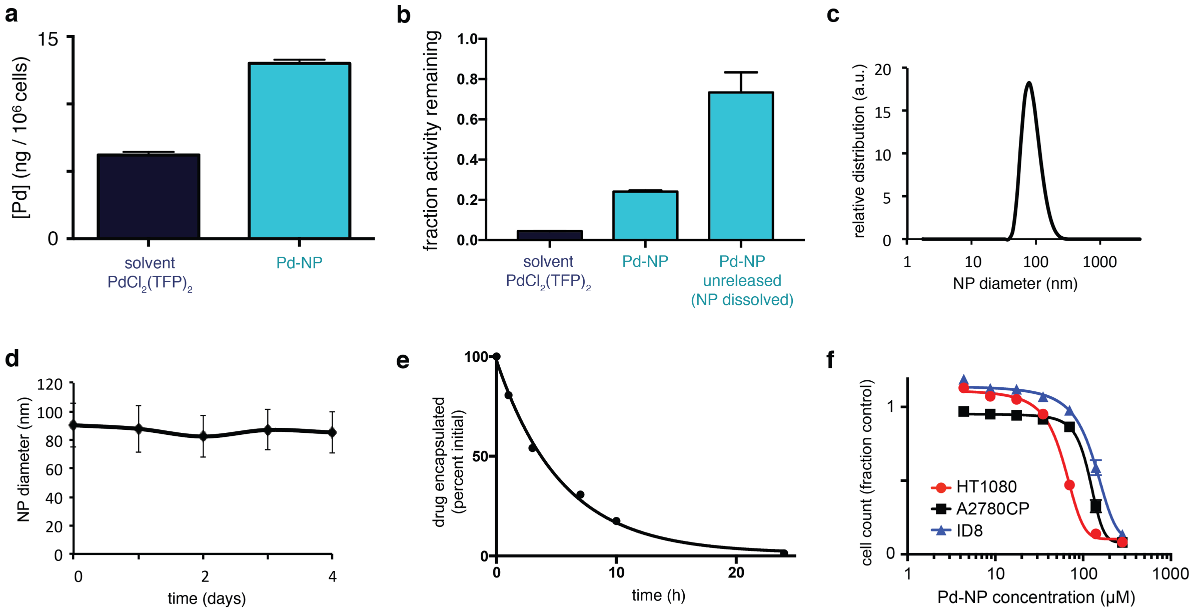


**Supplementary Figure 3.** ***In vitro*** **Pd-NP uptake, stability, toxicity, and Alloc$_2$R110 NP characterization.** **a)** ICP-MS measured intracellular [Pd] in HT1080 cell lysates after 24 h treatment with 25 μM Pd-NP or un-encapsulated $PdCl_2(TFP)_2$ catalyst (mean ± SEM, n = 2). **b)** Stability of $PdCl_2(TFP)_2$ after 24 h incubation in serum (FBS) was determined by un-caging of Alloc$_2$R110 (mean ± SD, n = 4). Activity of $PdCl_2(TFP)_2$ within Pd-NP, not yet released by 24 h incubation, was measured by first dissolving Pd-NP by adding 50% v/v DMF to the FBS solution. **c-e)** Characterization of Alloc$_2$R110 PLGA-PEG NP by DLS (*c*), stability of diameter in HBSS at 37$^{o}$C over time (*d*; mean ± SD, n = 3), and (*e*) *in vitro* release of Alloc$_2$R110 from NP encapsulation, measured in HBSS at 37$^{o}$C. **f)** Pd-NP cytotoxicity, measured by a resazurin-based assay after 72 h treatment across three cell lines (mean ± SEM; n=2).

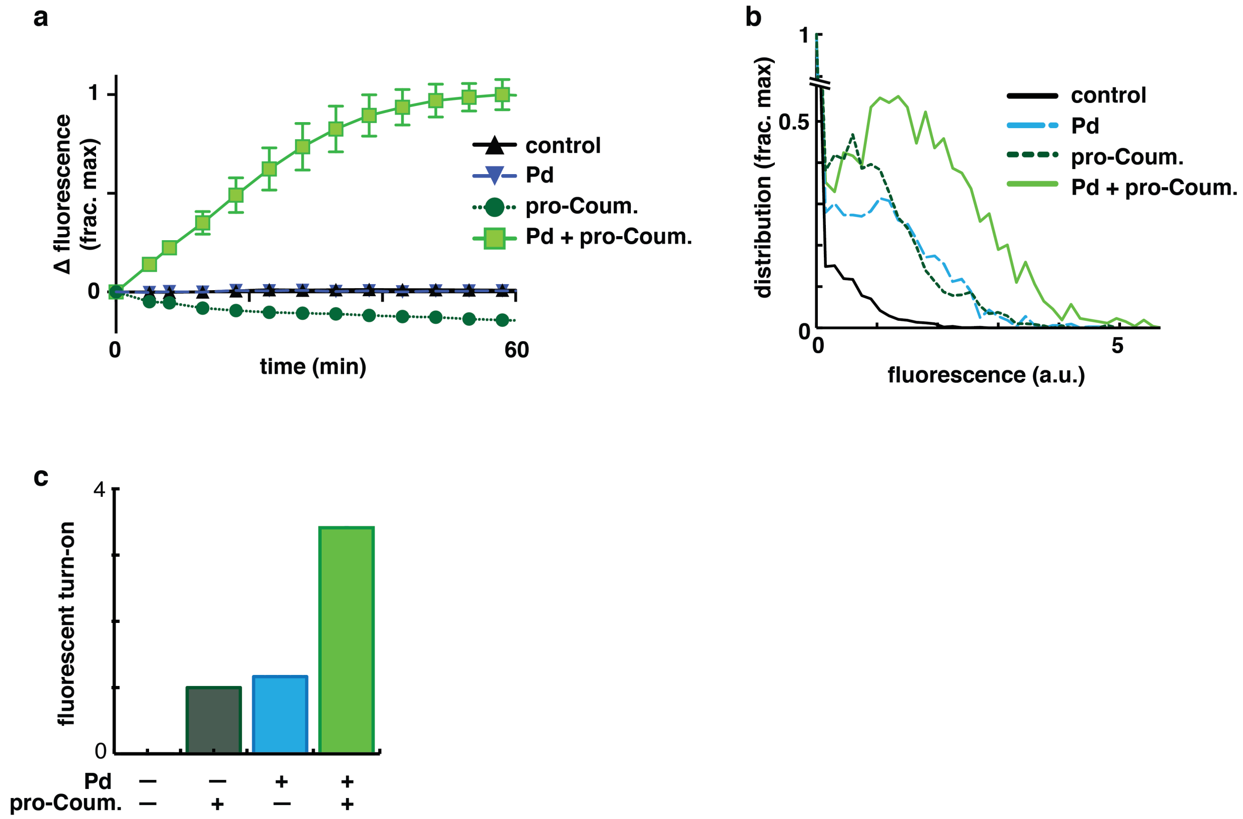


**Supplementary Figure 4. Kinetics and quantification of coumarin coupling.** **a)** Fluorescence increase of 30 µM coumarin precursor as it reacts with 10 µM $PdCl_2(TFP)_2$ in PBS at 37°C (mean ± SEM; n = 2). **b)** Single-cell distribution of intracellular coumarin fluorescence increase after 24 h reaction with 12.5 µM Pd-NP and 30 µM coumarin pre-cursor in full media at 37°C (n = 18). **c)** Intracellular fluorescence turn-on was calculated from data in *b* (mean ± SEM; n = 18).

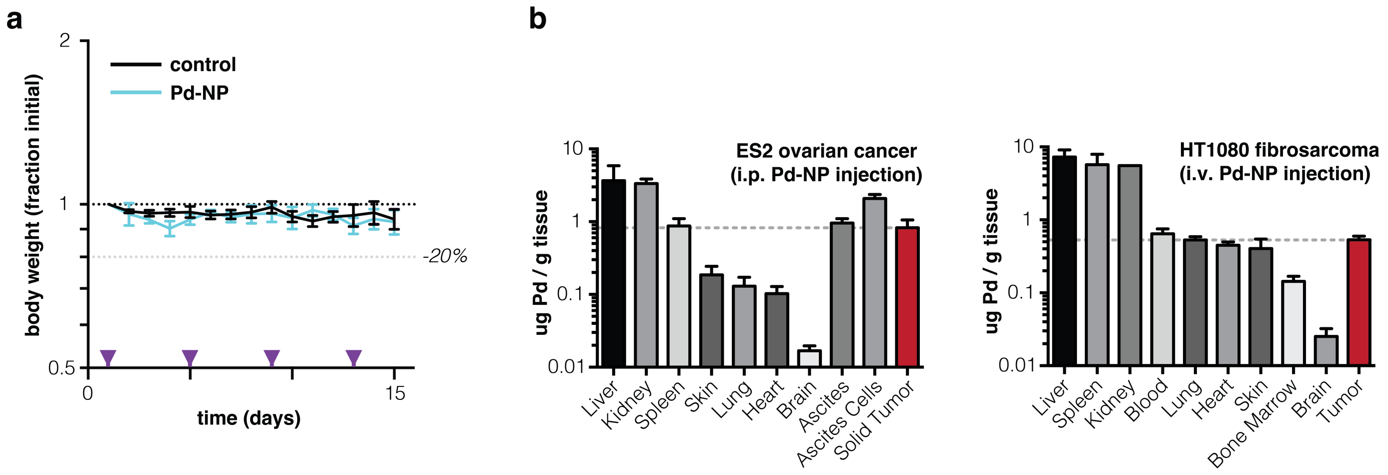


**Supplementary Figure 5. Pd-NP safely and efficiently accumulates in tumors. a)** Body weight in nu/nu mice bearing fibrosarcoma tumors monitored post-injection with either 50 mg $kg^{-1}$ Pd-NP or PLGA-PEG NP vehicle control (mean ± SEM, n = 6). Purple arrows mark treatment days. **b)** ICP-MS analysis of elemental Pd accumulation in tissue 24 h after 50 mg $kg^{-1}$ administration in two distinct tumor models (mean ± SEM, n=3).

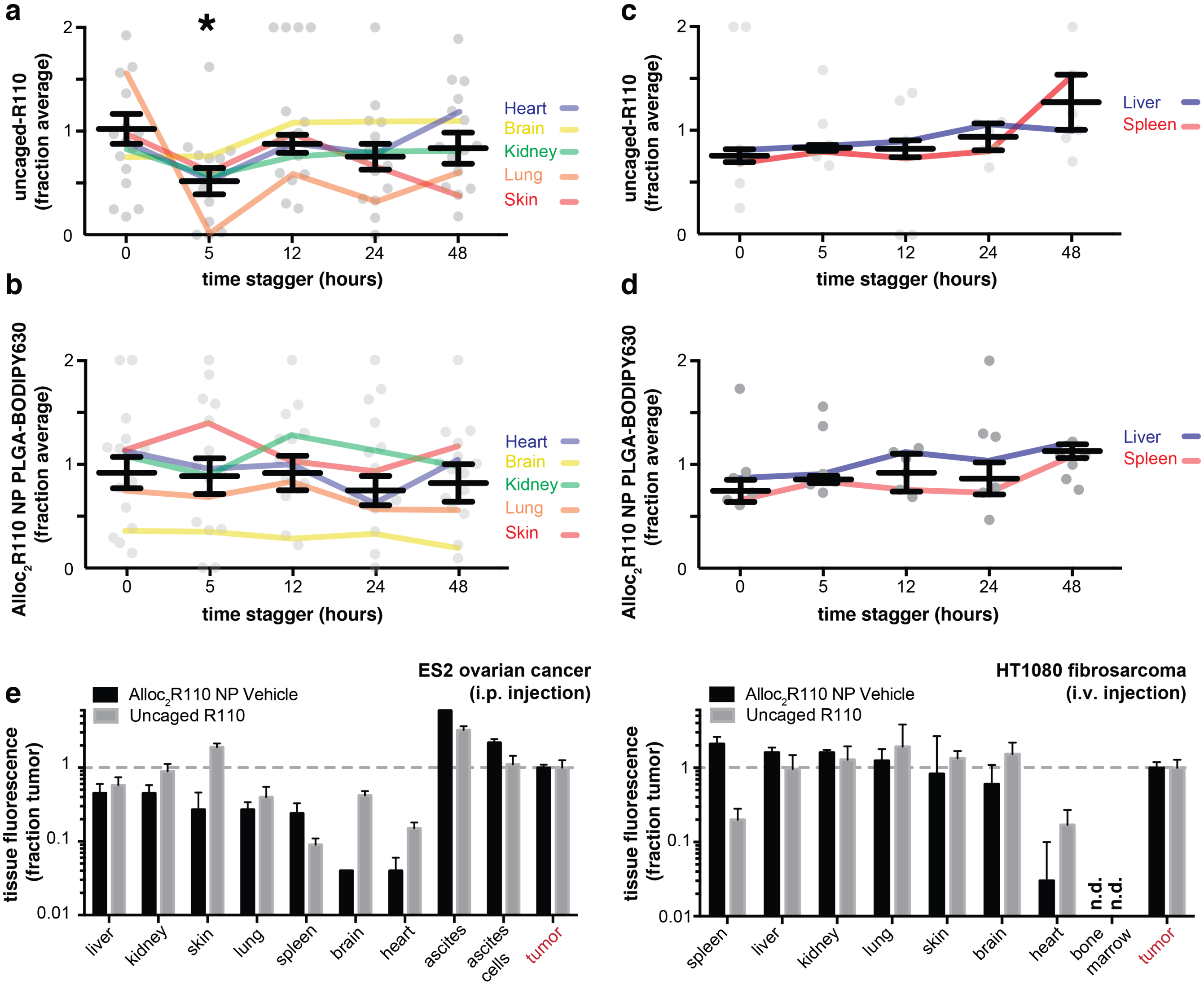


**Supplementary Figure 6. Time-staggered administration of Pd-NP and its substrate leads to tumor-selective activity. a-d)** In the fibrosarcoma tumor model, Pd-NP was first injected followed by NIR-labeled Alloc$_2$R110 NP at the indicated time points, and 24 h later organs were excised and analyzed by fluorescence for R110 uncaging (*a-b*) and accumulation of the Alloc$_2$R110 NP vehicle (*c-d*). Data are shown relative to fluorescence in the tumor for each animal and across all replicates and time-points for each organ, such that each dot represents one organ measurement, lines denote averages across n=3 replicates for each organ, and bars denote mean ± SEM (n = 15) across all organs (*p=0.003, two-tailed t-test). **e)** 5 h time-staggering and tissue analysis was performed as described above (see *a-d*), using i.p. injections in the ES2 orthotopic OVCA model and i.v. injections in the HT1080 fibrosarcoma model; n.d., not detected (mean ± SEM, n=3).

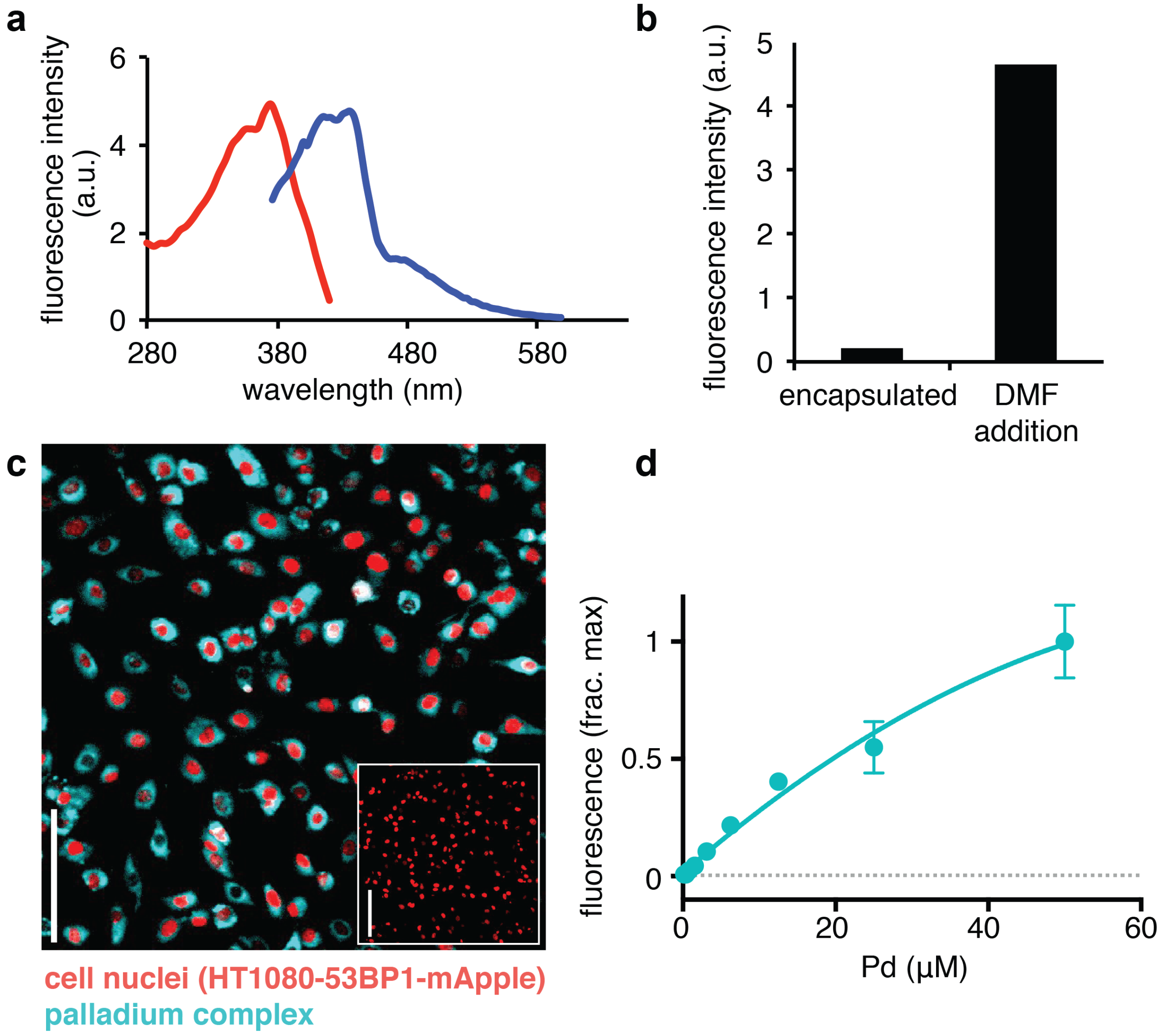


**Supplementary Figure 7. Fluorescent properties of Pd-NP. a)** Fluorescence excitation and emission spectra of $PdCl_2(TFP)_2$ in HBSS/DMSO. **b)** Pd nano-encapsulation quenches fluorescence, which increases 36-fold upon dissolution of the nanoparticle via DMF addition. **c)** Fluorescent microscopy reveals intracellular fluorescence generated by the 25 µM Pd-NP within cancer cells following 24 h treatment. Inset: untreated control. Scale bar, 100 µm. **d)** Intracellular fluorescence from 24 h Pd-NP treatment increases with dose, measured by microscopy in HT1080 cells (mean ± SEM, n = 9).

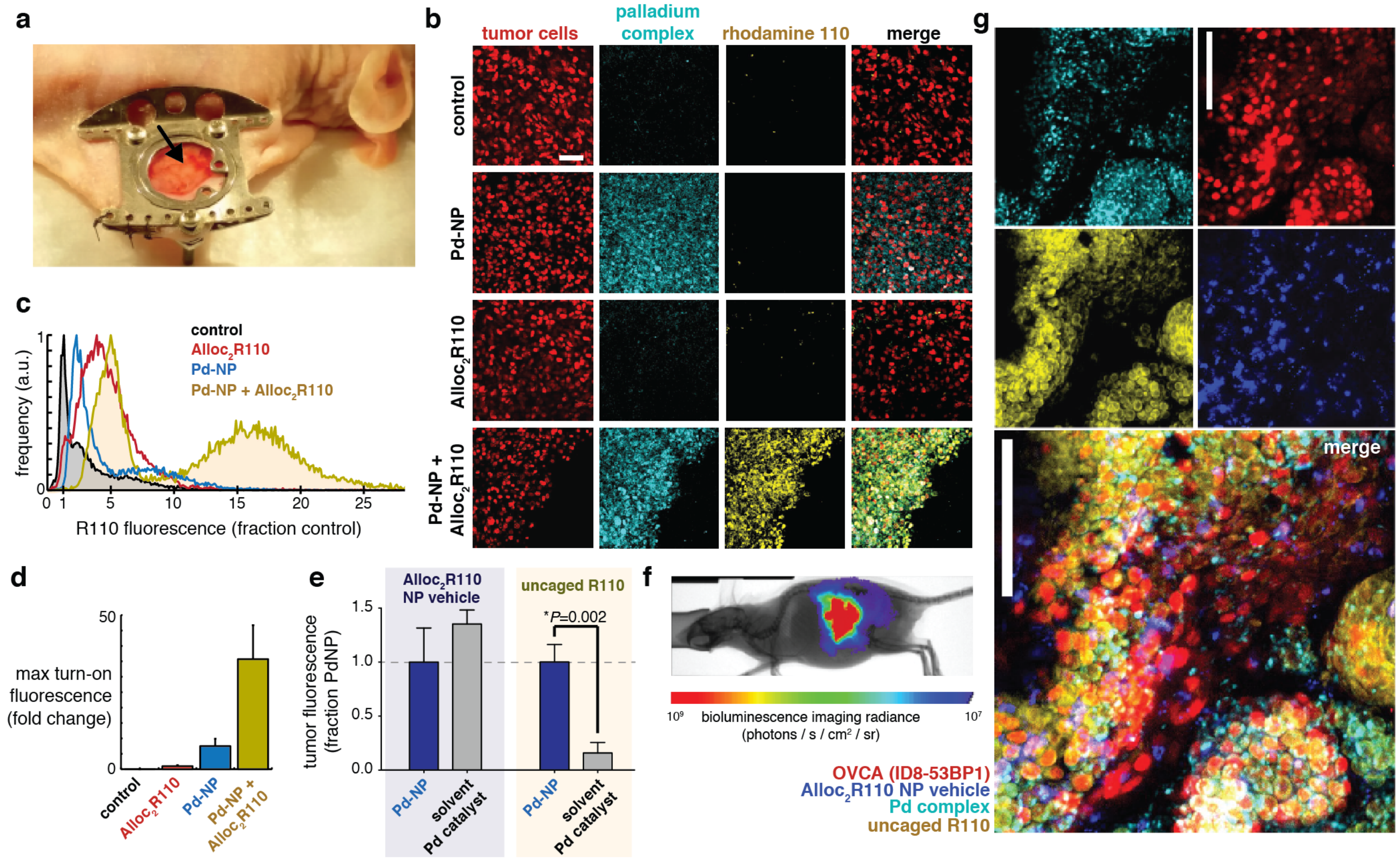


**Supplementary Figure 8. Intratumoral Alloc$_2$R110 turn-on in window chamber and orthotopic xenografts. a)** Dorsal skinfold window chamber showing implanted HT1080-53BP1-mApple xenograft tumor (arrow). **b-d)** HT1080 tumors were treated with 25 mg kg$^{-1}$ Alloc$_2$R110 NP 5 h following treatment with 50 mg kg$^{-1}$ Pd-NP by i.v. injection. 24 h later tumors were excised, imaged (*b;* scale bar, 100 µm), quantified according to single-cell fluorescence distribution using automated image segmentation (*c*), and from imaging data, max fluorescence turn-on values were computed and averaged across n = 4 tumors (*d*; mean ± SEM). **e)** The same experiment as in *b-d* was performed, but using un-encapsulated Pd complex in a solvent formulation rather than Pd-NP. Alloc$_2$R110 NP were labeled with a NIR dye to quantify their tumor uptake (which did not depend on Pd nano-encapsulation, left), and un-caging was monitored by fluorescence of excised tissue and normalized to fluorescence observed when using Pd-NP (mean ± SEM, n=4, two-tailed t-test). **f)** Bioluminescence imaging of the orthotopic and syngeneic model of ovarian cancer using intrabursally implanted ID8 tumors transgenically expressing luciferase. **g)** Corresponding with (*f*)**,** Pd-NP activity was measured in the ID8 model. 50 mg kg$^{-1}$ Pd-NP was injected, followed by 25 mg kg$^{-1}$ Alloc$_2$R110 with NIR-labeled NP vehicle 5 h later. Intraperitoneal metastases were excised 24 h later and immediately imaged (representative from n = 3 tumors). Scale bar, 100 µm.

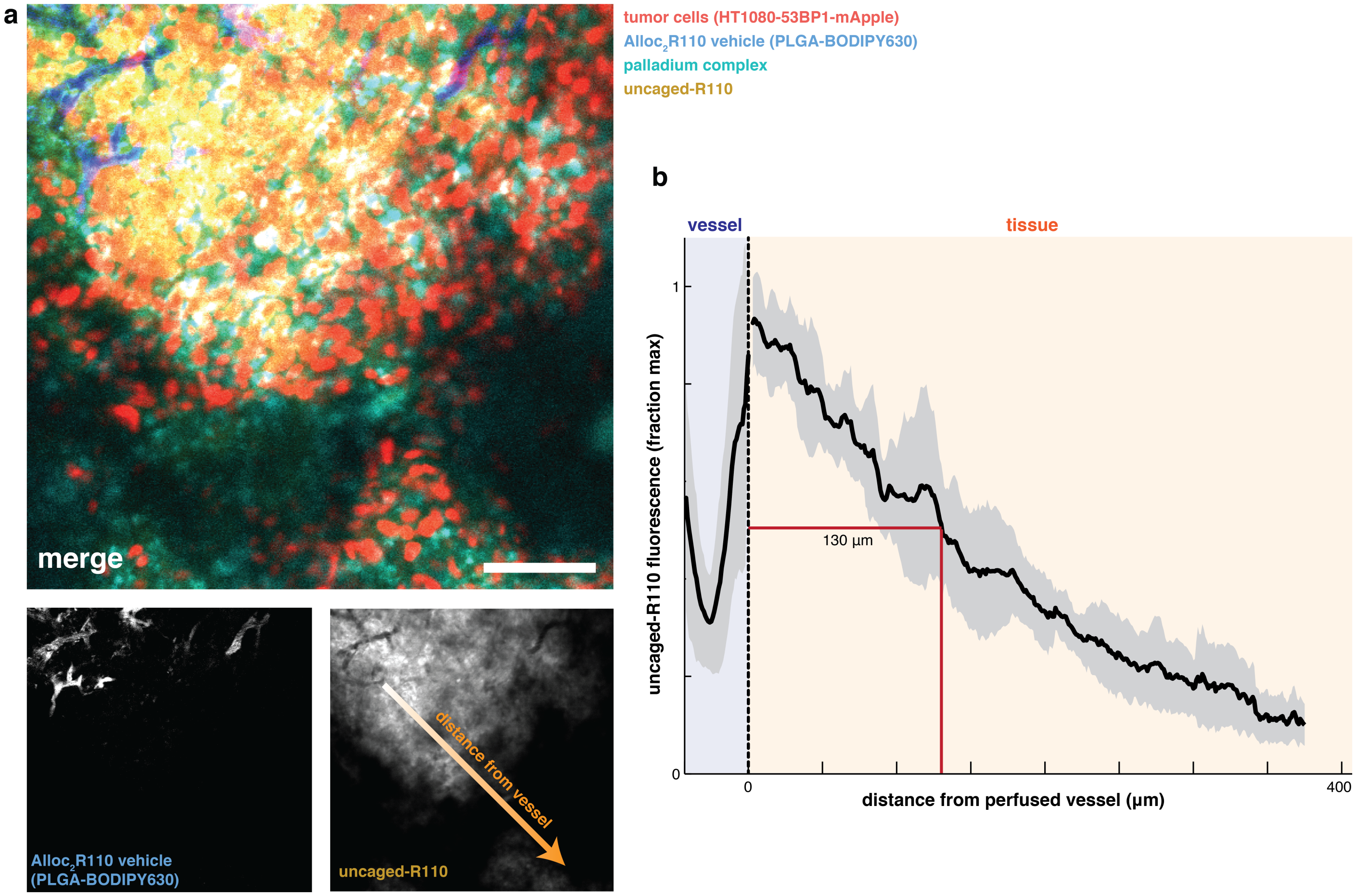


**Supplementary Figure 9. Local Pd catalyst activity depends on proximity to tumor microvasculature.** HT1080 tumors were treated with 25 mg $kg^{-1}$ $Alloc_2R110$ NP 1 h following treatment with 50 mg $kg^{-1}$ Pd-NP by i.v. injection. After 2 h post-injection with $Alloc_2R110$, tumors were imaged for co-localization between microvasculature (still visible by circulating $Alloc_2R110$ NP), Pd complex, and uncaging of its substrate. **b)** Profiles of fluorescence intensity were measured as a function of distance to the nearest vasculature, focusing on local tumor regions that lacked substantial vasculature identified by 3D z-stack image datasets. Thick line and shading denote mean ± SEM, respectively (n=7). Scale bar = 100 µm.

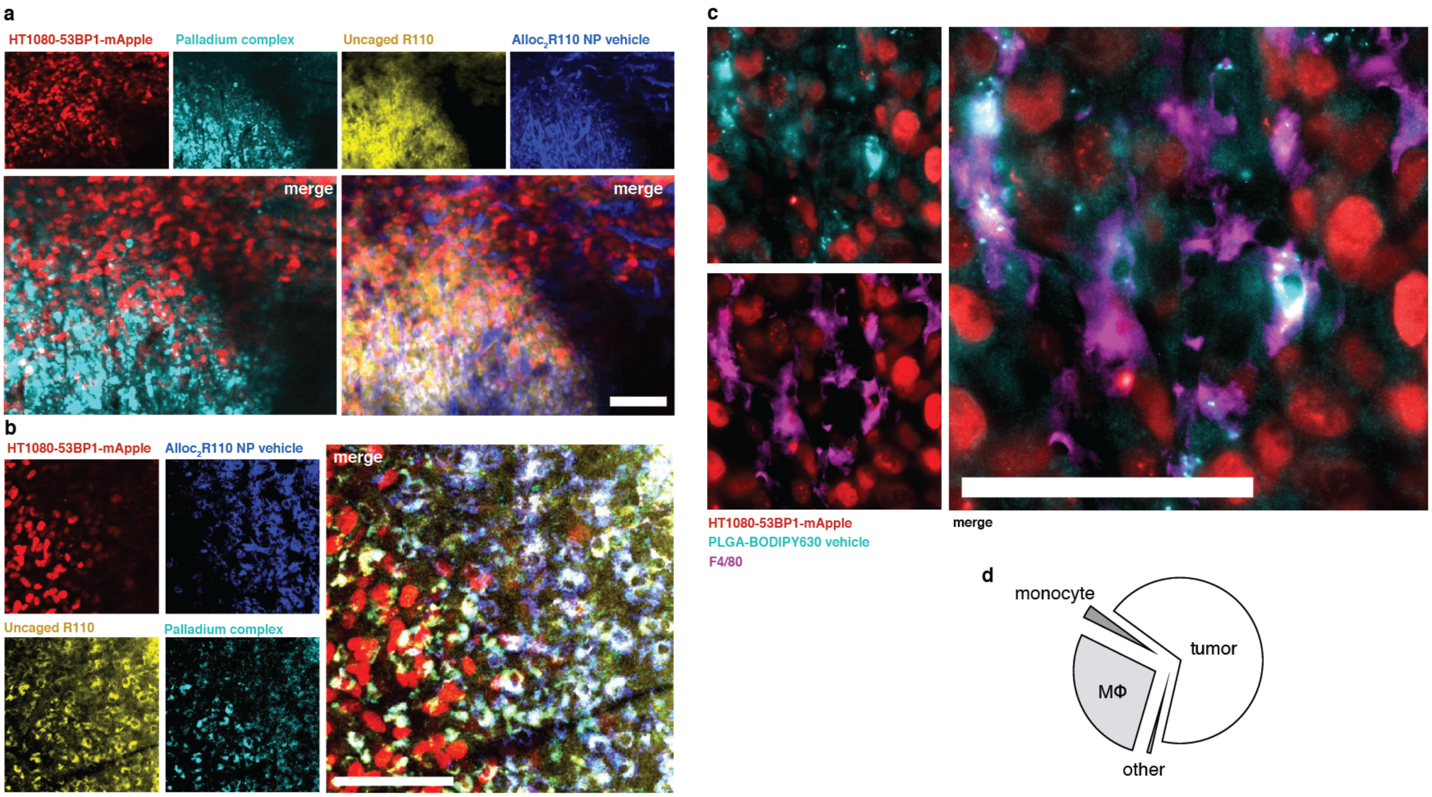


**Supplementary Figure 10. Tumor-associated host phagocytes accumulate Pd-NP. a-b)** Pd-NP treatment followed by injection of $Alloc_2R110$ NP with NIR-labeled NP vehicle (via PLGA-BODIPY630 co-encapsulation) leads to local Pd-NP activity in both tumor cells and tumor-associated host phagocytes 3 h (a) and 24 h (b) following injection (n ≥ 3). Scale bar, 100 µm. **c)** Immunofluorescence of NIR-labeled NP vehicle 24 h post-injection shows high NP accumulation in tumor cells and F4/80+ host phagocytes in HT1080-53BP1-mApple xenograft tumors (n = 4). Scale bar, 50 µm. **d)** FACS analysis (n ≥ 4) of NIR-labeled NP vehicle 24 h post-injection shows NP uptake primarily in tumor cells (HT1080), MΦ ($CD45^+$ $CD11b^+$ $Lin^-$ $Ly6C^-$ $F4/80^+$ $CD11c^+$), with little additional accumulation in monocytes and other cell populations (including lymphocytes, neutrophils, along with $CD45^-$ fibroblasts, pericytes, endothelium, and other stromal populations). Graph shows distribution of total NP within the bulk tumor as a function of cell-type.

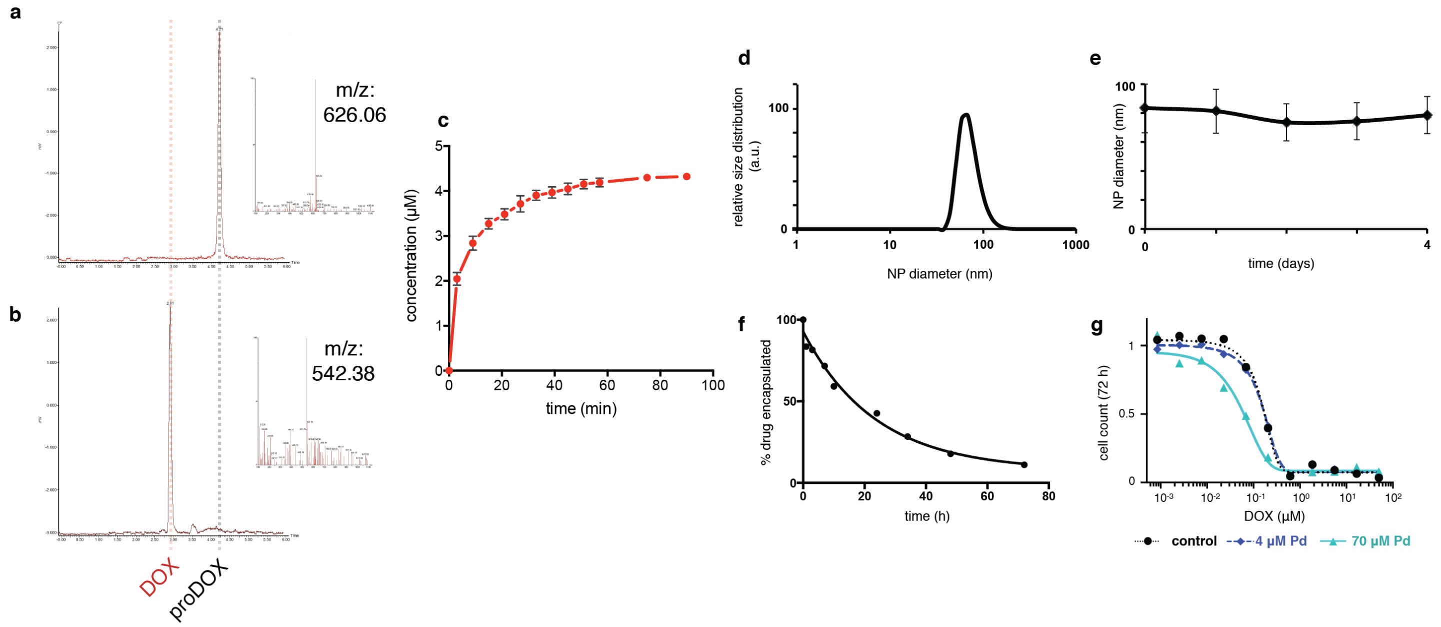


**Supplementary Figure 11. Characterization of proDOX activation mediated by Pd-NP. a-b)** 10 μM Pd-NP was incubated with 10 μM proDOX NP for 24 h in HBSS at 37$^o$C, and was analyzed by LC/MS before (a) and after (b) incubation. Plots at left show fluorescence detection as a function of elution time, while plots at right show corresponding HR-ESI-MS (negative mode) mass fragmentation patterns of the indicated peaks for proDOX ($C_{31}H_{32}NO_{13}$: m/z [M -H]$^-$ calcd: 626.20; obsd: 626.06) and DOX ($C_{27}H_{28}NO_{11}$: m/z [M -H]- calcd: 542.17; obsd: 542.38). **c)** 10 μM $PdCl_2(TFP)_2$ was incubated with 5 μM proDOX in HBSS at 37$^o$C, and proDOX conversion to DOX was monitored by HPLC fluorescence chromatography (means ± SEM, n = 2). **d)** DLS-measured distribution of proDOX-encapsulated PLGA-PEG NP according to diameter. **e)** ProDOX NP is stable in HBSS at 37$^o$C over time (mean ± SD, n = 3). **f)** *In vitro* release of proDOX from NP encapsulation, measured in HBSS at 37$^o$C. **g)** *In vitro* cell count (measured by resazurin-based assay) of HT1080 after 72 h treatment with varying doses of Pd-NP and DOX-NP (as compared to proDOX-NP co-treatment, Fig. 4). Data are means ± SEM, n = 2.

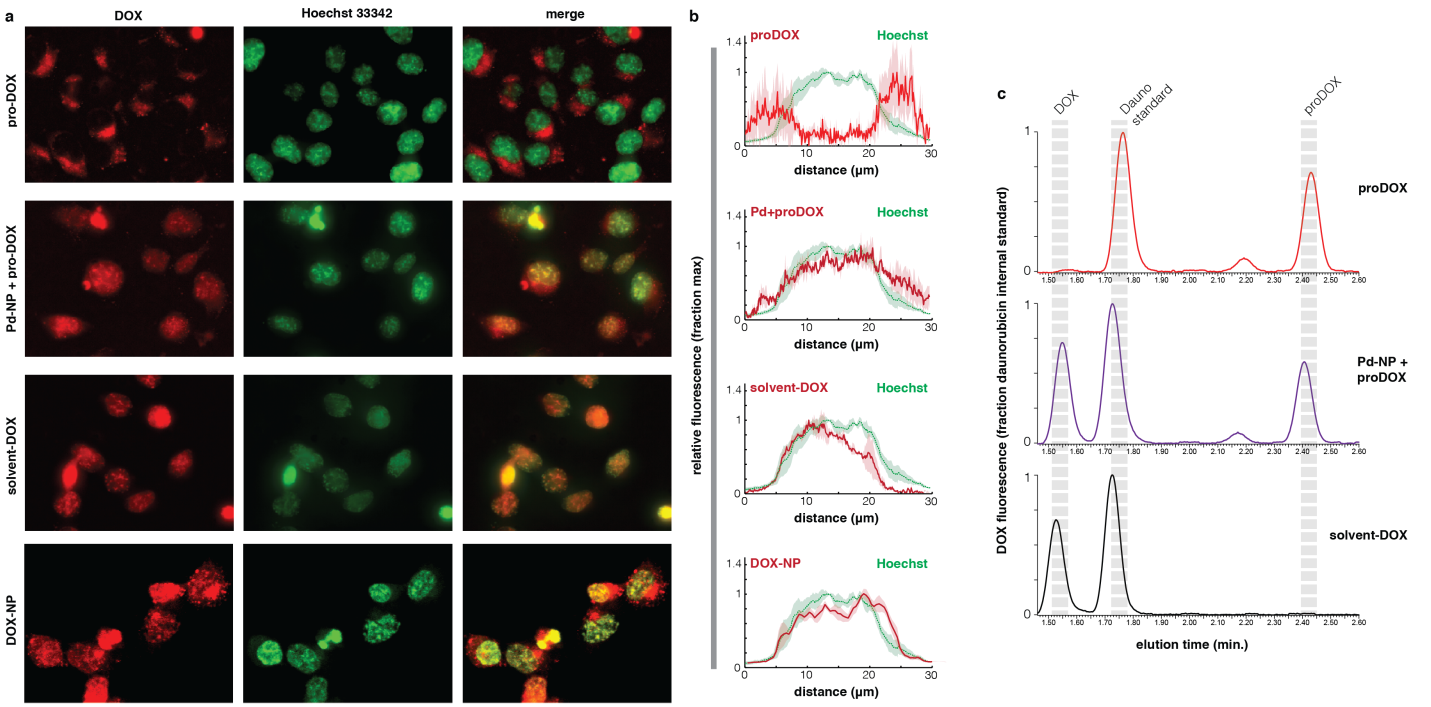


**Supplementary Figure 12. Characterization of proDOX activation by Pd-NP in cell culture. a)** Endogenous DOX and proDOX (1 µM) fluorescence was imaged in ES2 OVCA cells after 16 h incubation, in the presence or absence of 30 µM Pd-NP co-treatment, after washing and DNA counter-staining with Hoechst 33342. **b)** Corresponding to images in *a*, average cell profiles of hoechst and drug fluorescence were quantified to determine selective nuclear accumulation of proDOX only after Pd-NP co-treatment (lines and shading denote means ± SEM, n>10). **c)** Co-treatment of proDOX (1 µM) with Pd-NP (30 µM) leads to 51% drug activation in HT1080 cells after 12 h treatment, determined after washing, lysing, and analyzing using MeCN extraction and HPLC fluorometric chromatography.

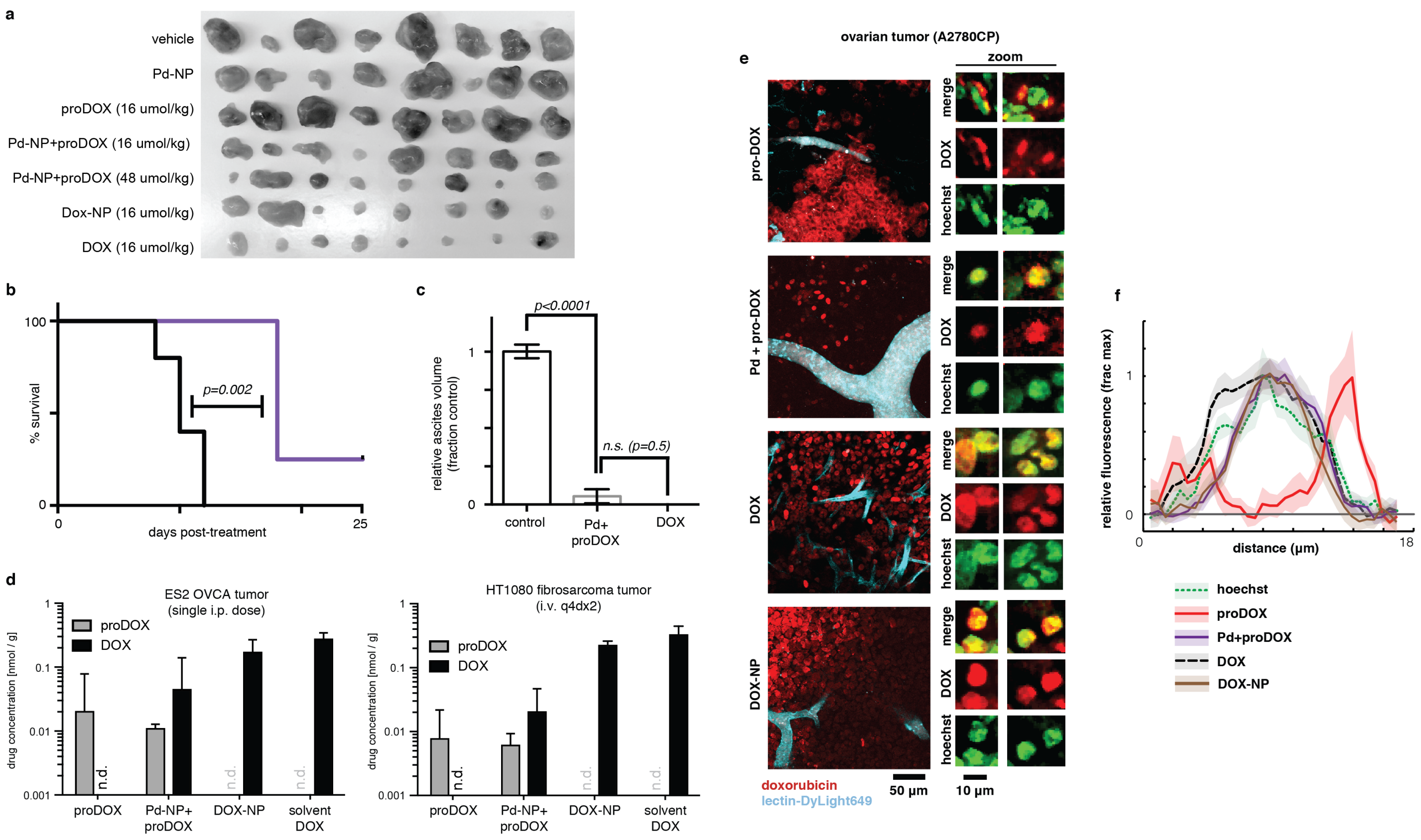


**Supplementary Figure 13. Nano-palladium efficacy in fibrosarcoma and OVCA tumor models. a)** Representative images of excised HT1080 tumors after 8 days treatment (see Fig. 5d). **b-c)** Human ES2 ovarian cancer cells were administered intraperitoneally and 3 days later were treated with either the drug-free vehicle or the combination of Pd-NP followed by proDOX NP. **b)** Combination Pd-NP and proDOX treatment (purple line) extends survival compared to the vehicle control (black line) ($p = 0.002$, log-rank test, $n \geq 4$). **c)** 11 days post-treatment in the ES2 intraperitoneal xenograft, the control group developed significant ascites while the treatment groups did not (data are means ± SEM; two-tailed t-test, $n = 4$). **d)** proDOX and activated DOX were quantified by HPLC from two different xenograft models, 24 h post-treatment (mean ± 95% C.I.; $n \geq 3$; n.d. = not detected). **e)** Pre-treatment with Pd-NP followed by proDOX leads to drug accumulation in tumor nuclei of orthotopic A2780CP ovarian tumors. 24 h post-treatment, tumors were excised and imaged for co-localization between intrinsic doxorubicin fluorescence and DNA (via Hoechst 33342 counterstain); lectin marks tumor vasculature. **f)** Traces show mean (thick line) ± SEM (shading) in single-cell profiles of drug fluorescence across $n \geq 4$ tumors.

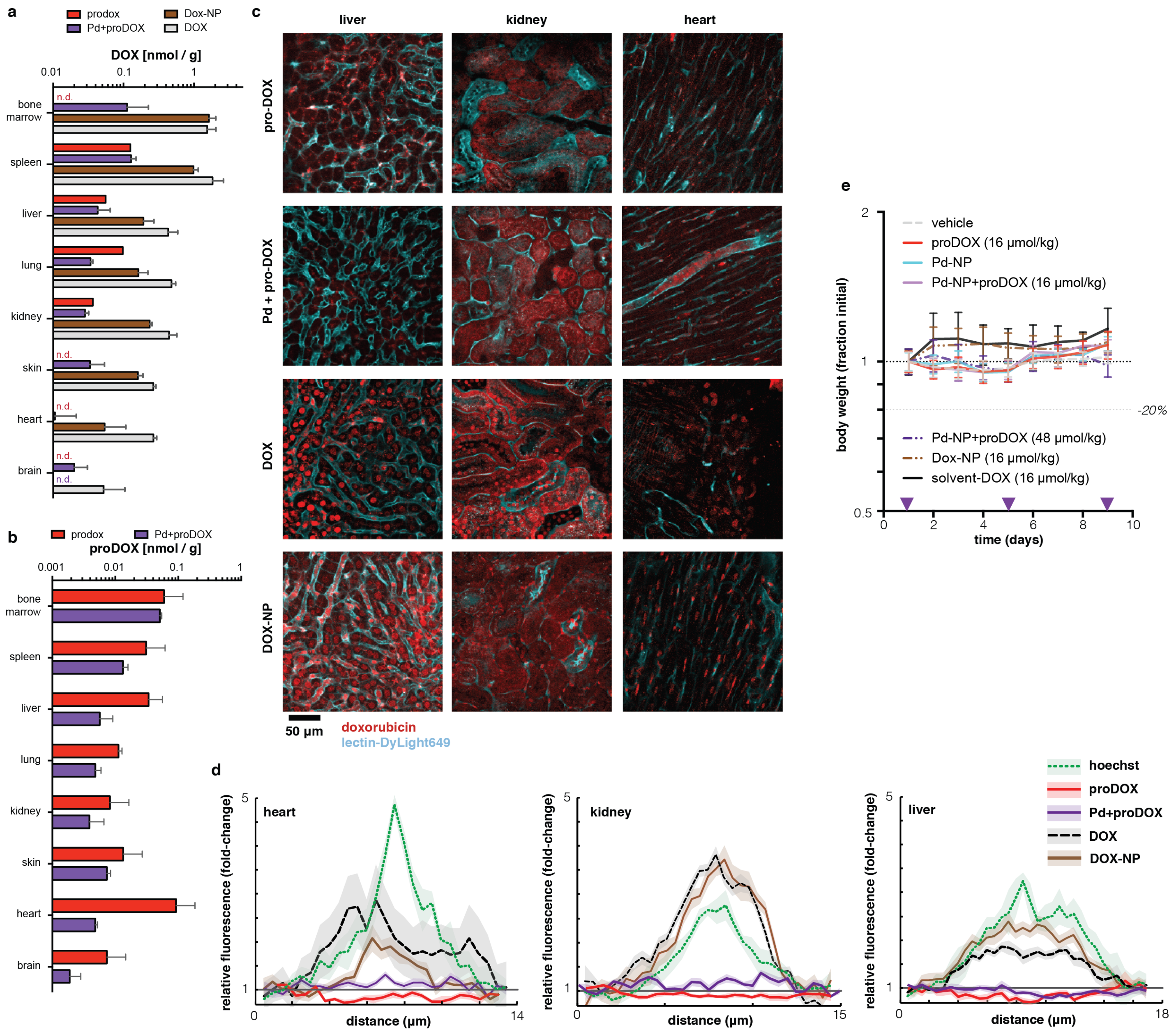


**Supplementary Figure 14. Selective proDOX activation minimizes systemic drug exposure. a-b)** 24 h following q4dx2 i.v. treatments in the fibrosarcoma mouse model, organs were excised and analyzed for DOX (*a;* n = 3) and proDOX (*b; n* ≥ 2) concentrations using HPLC (mean ± SEM; n.d., not detected). **c-d)** 24 h after i.p. treatment, organs were excised from OVCA-bearing mice and imaged for co-localization between intrinsic doxorubicin fluorescence (red) and DNA via Hoechst 33342 counterstain (n ≥ 4); lectin-DyLight649 labels vasculature (light blue). Data show organs relevant to drug toxicity. **d)** Using data as in *c*, drug accumulation in nuclei of key organs related to toxicity was quantified based on Hoechst co-localization. Traces show mean (thick line) ± SEM (shading) across populations of single cells in n ≥ 4 tumors (corresponds to Fig. 6b-c). **e)** No treatment caused significant loss in body weight in nu/nu mice bearing HT1080 tumors (α = 0.05; n ≥ 5; two-tailed t-test). Purple arrows mark treatment days.

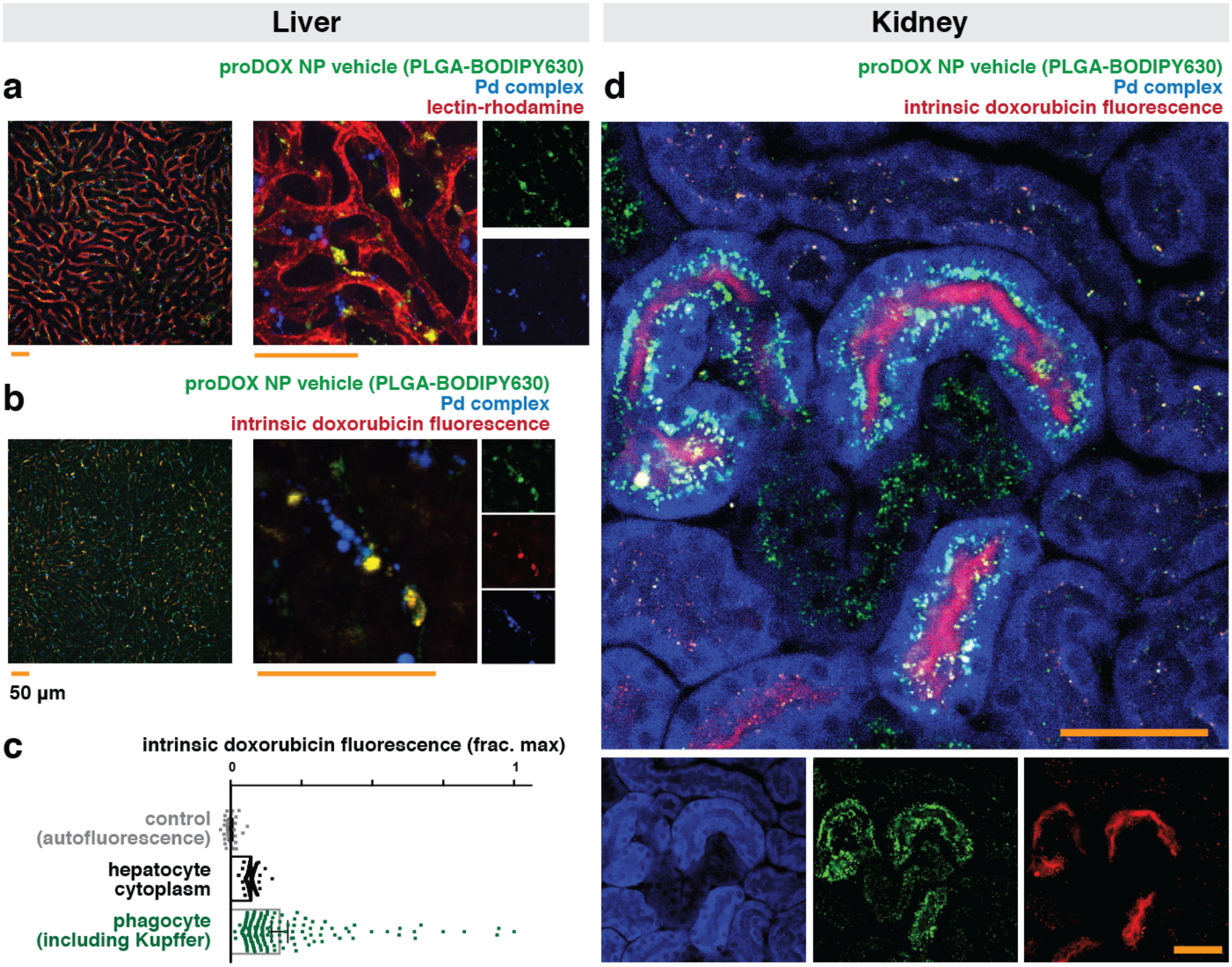


**Supplementary Figure 15. Imaging suggests mechanisms of mitigated off-target prodrug activation in clearance organs. a)** 24 hr following Pd-NP treatment and 19 hr following treatment with NIR-labeled proDOX NP vehicle, nu/nu mice were injected with lectin-rhodamine to label vasculature. 5 min later, livers were excised and imaged for patterns of Pd complex accumulation (imaged by its intrinsic fluorescence), proDOX NP vehicle, and vasculature. Results show accumulation in perivascular phagocytes (which can include resident liver macrophage, Kupffer cells) rather than larger, morphologically distinct hepatocytes. **b)** The same experiment was performed as in *a*, but with imaging intrinsic fluorescence of proDOX-encapsulated NP (10 mg $kg^{-1}$) rather than lectin-rhodamine. Results show co-accumulation of proDOX, the proDOX NP vehicle, and Pd complex in similar phagocyte populations. However, the intra-phagocyte distribution of Pd complex appears distinct from proDOX at the subcellular level (Pearson's correlation coefficient after thresholding = -0.12 ± 0.3, mean ± SD, n=3). **c)** ProDOX accumulates more in perivascular phagocytes of the liver compared to morphologically distinct hepatocytes. **d)** Kidneys were imaged similarly to the liver in *b*, showing distinct localization of Pd complex and its substrate proDOX (Pearson's correlation coefficient after thresholding = -0.2 ± 0.02, mean ± SD, n=3).

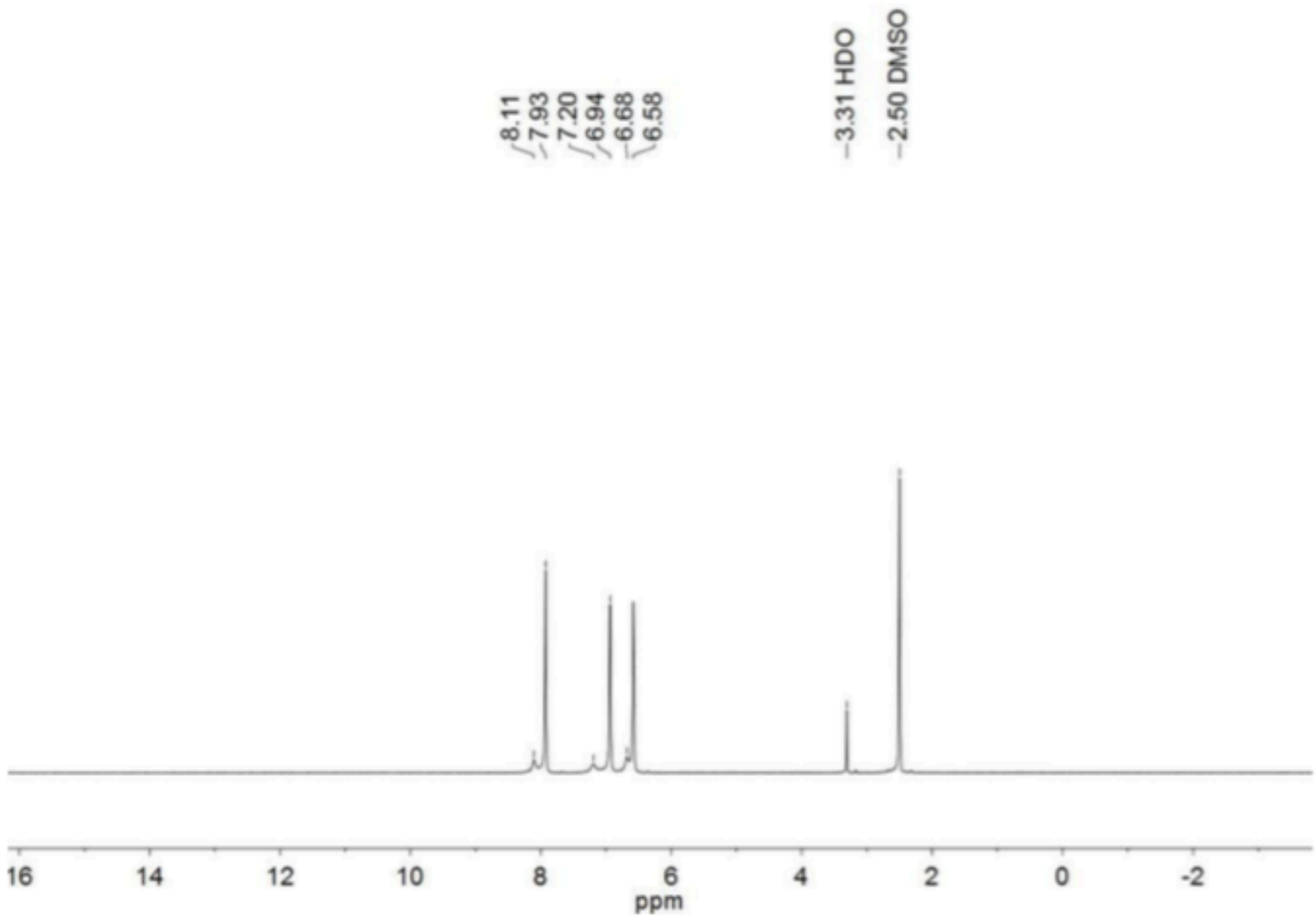


**Supplementary Figure 16.** $^1$H NMR spectrum (DMSO-d6, r.t.) of compound **1**.

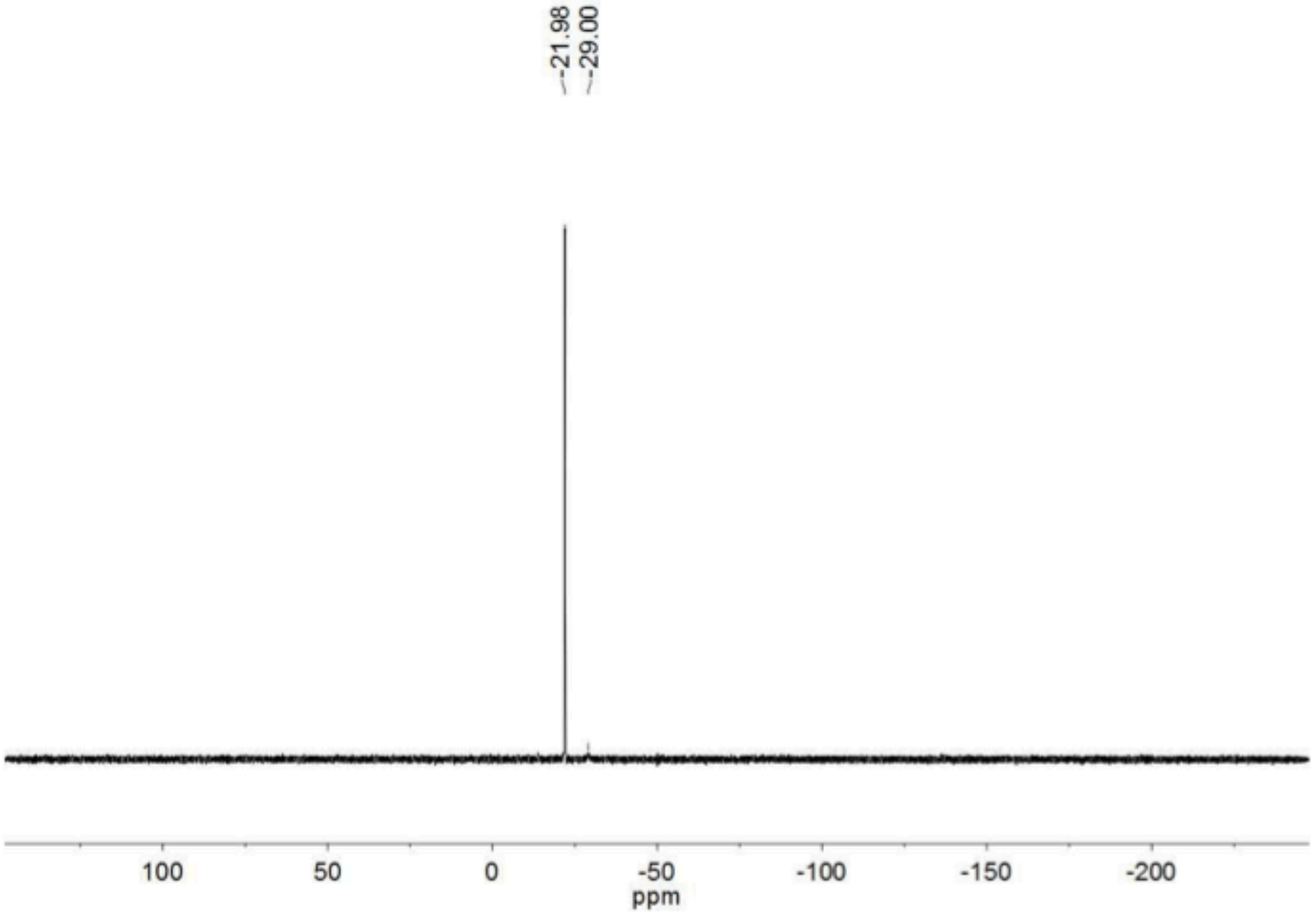


**Supplementary Figure 17.** $^{31}P$ NMR spectrum (DMSO-d6, r.t.) of compound **1**.

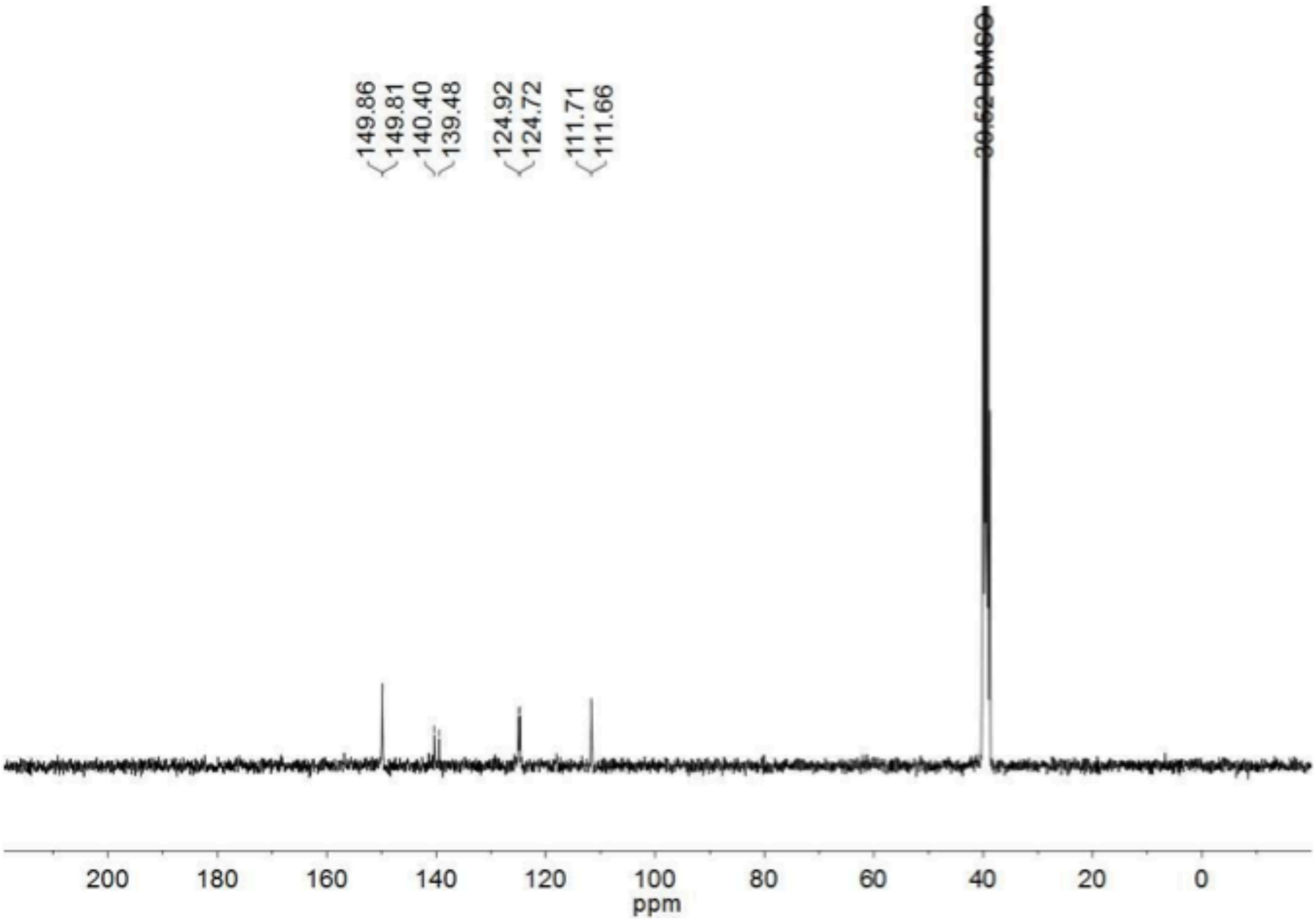


**Supplementary Figure 18.** $^{13}C$ NMR spectrum (DMSO-d6, r.t.) of compound **1**.

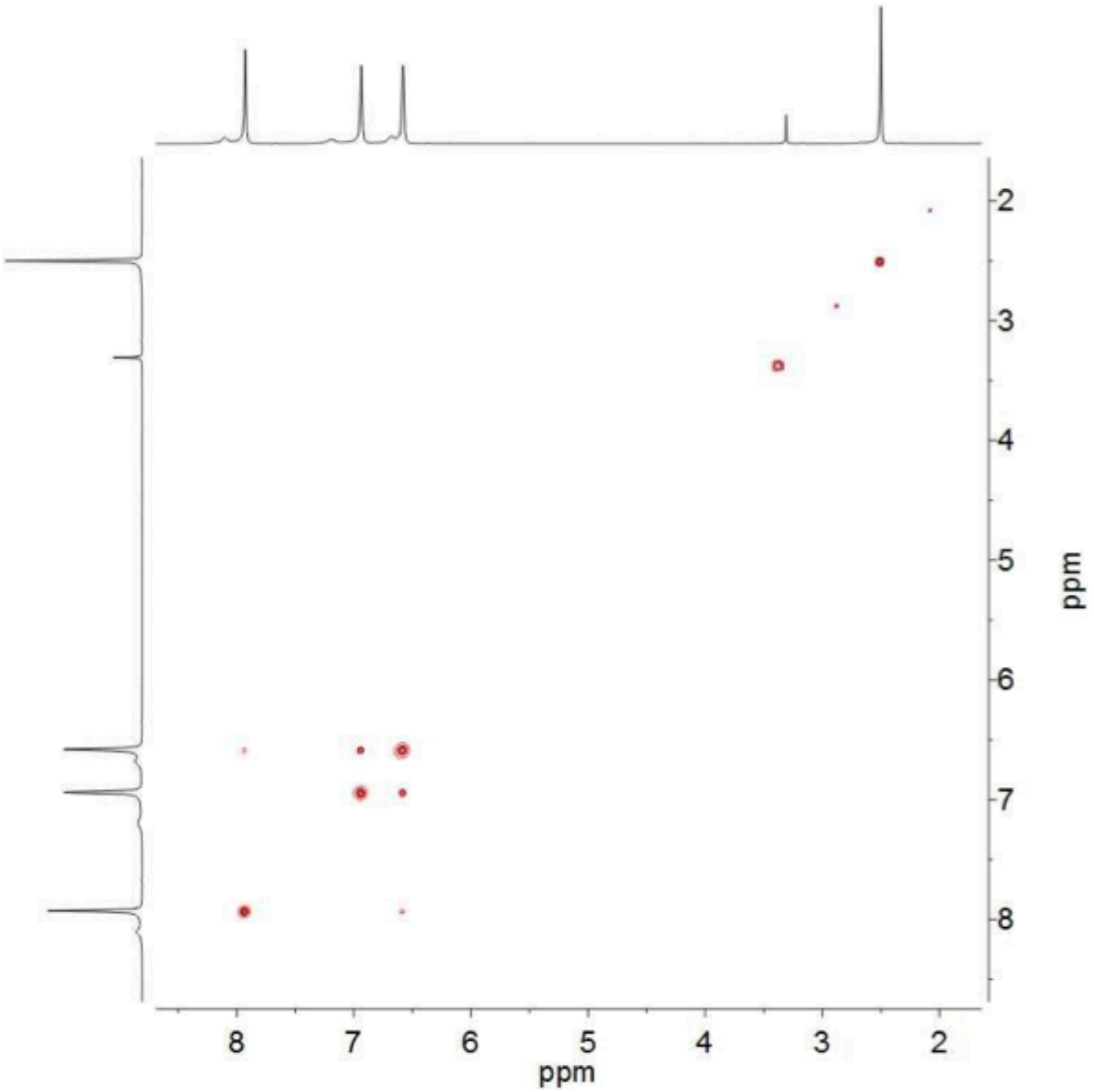


**Supplementary Figure 19.** $^1$H COSY NMR spectrum (DMSO-d6, r.t.) of compound **1**.

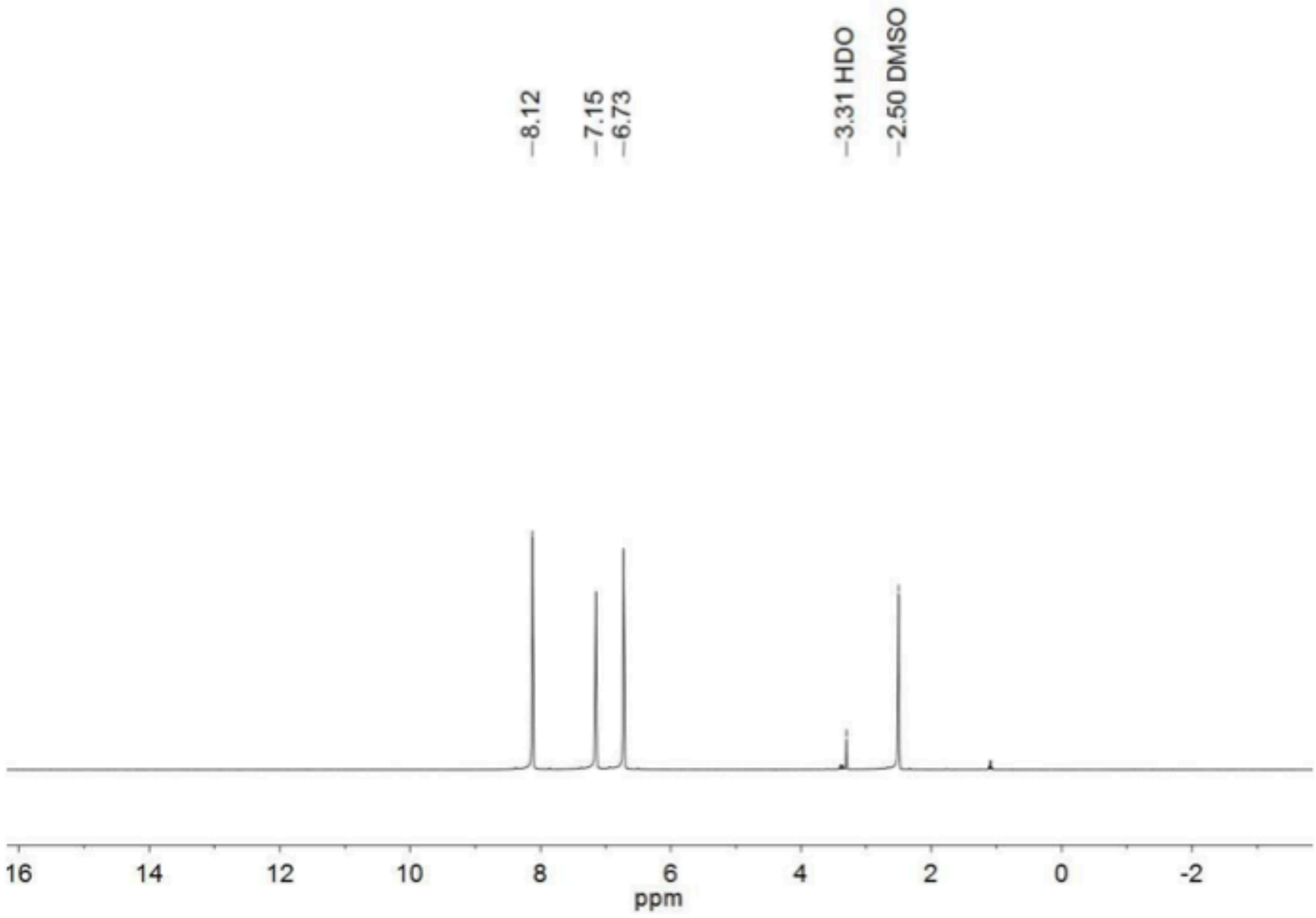


**Supplementary Figure 20.** $^{1}$H NMR spectrum (DMSO-d6, r.t.) of tri(2-furyl)phosphine oxide.

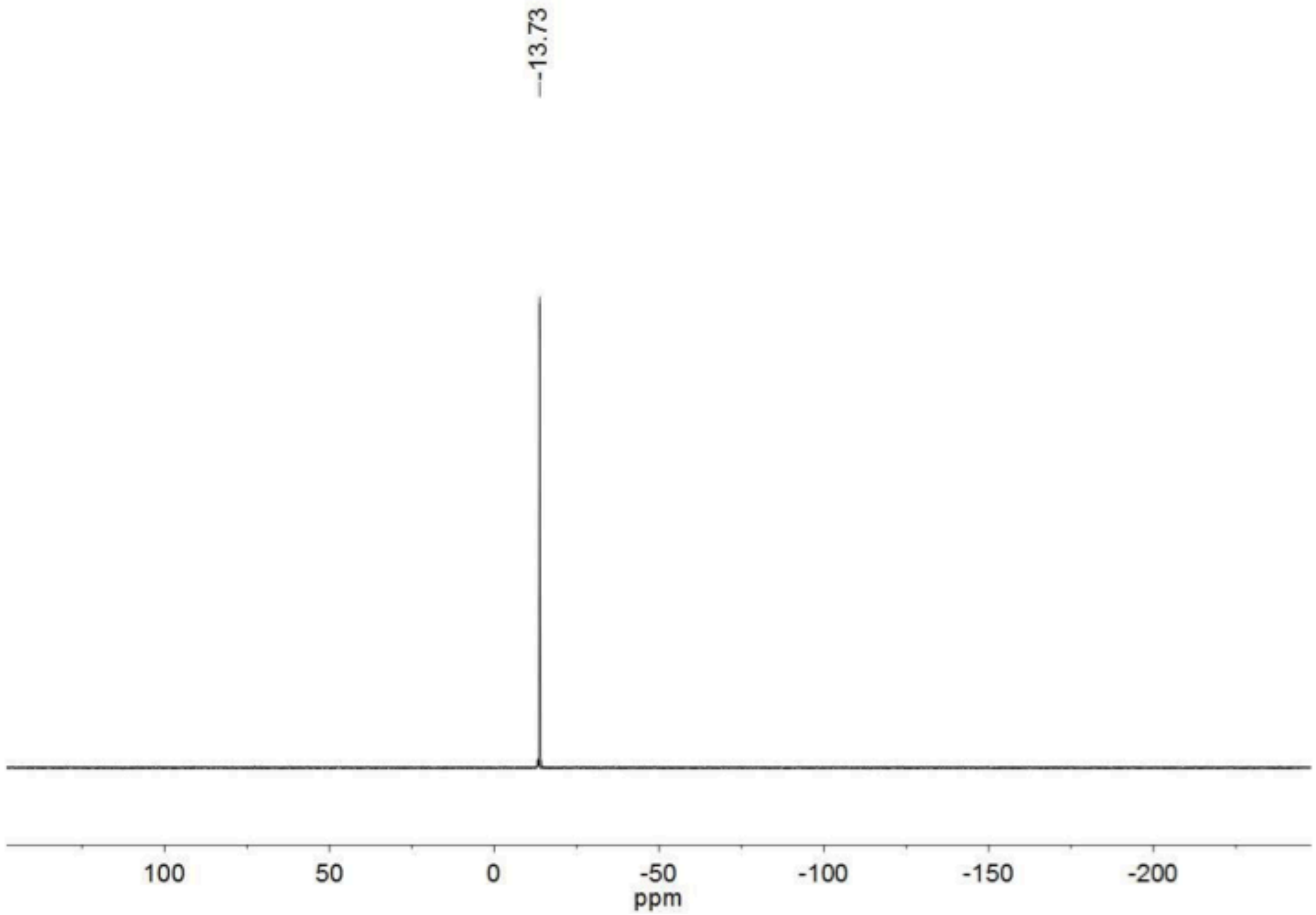


**Supplementary Figure 21.** $^{31}P$ NMR spectrum (DMSO-d6, r.t.) of tri(2-furyl)phosphine oxide.

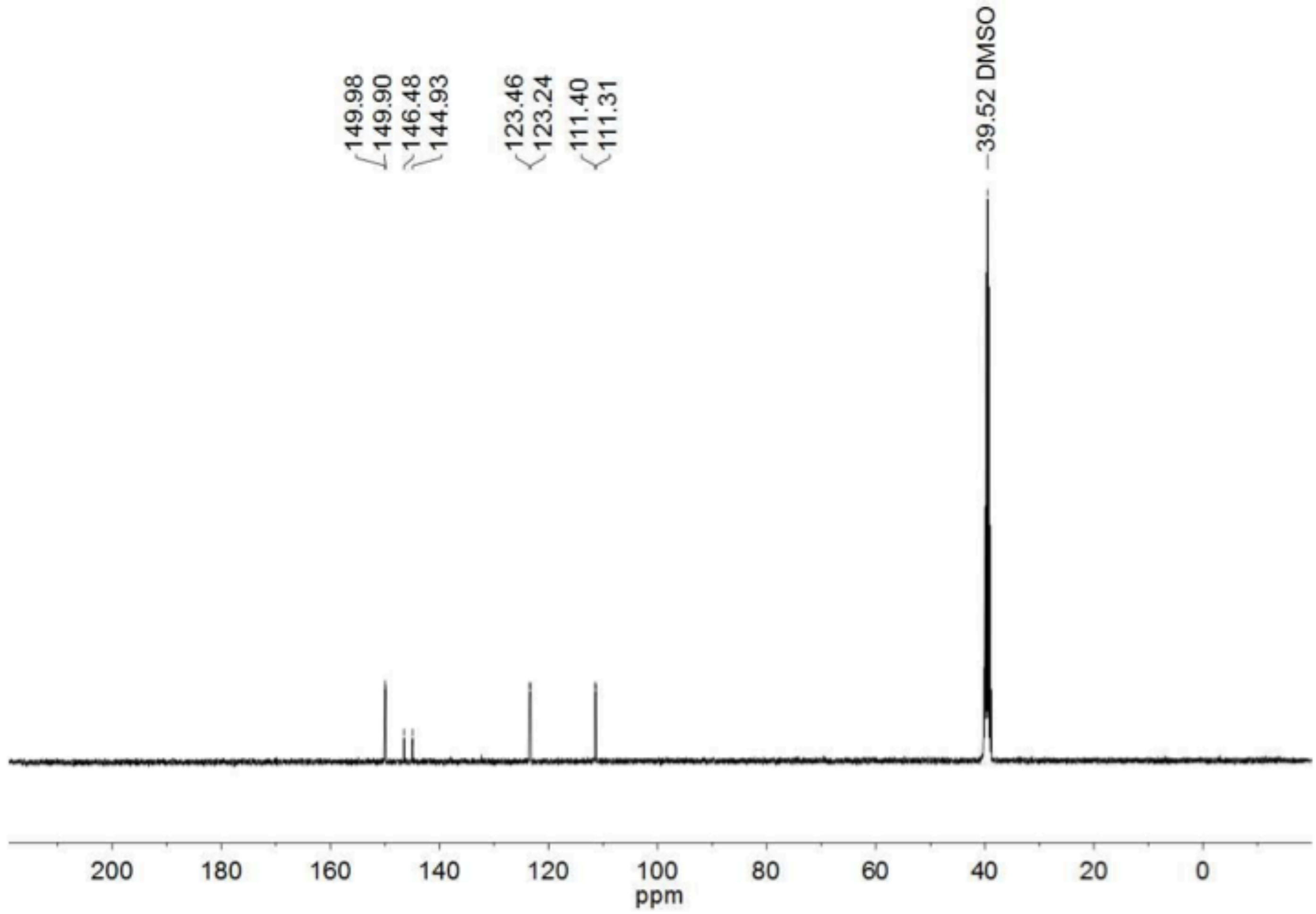


**Supplementary Figure 22.** $^{13}C$ NMR spectrum (DMSO-d6, r.t.) of tri(2-furyl)phosphine oxide.

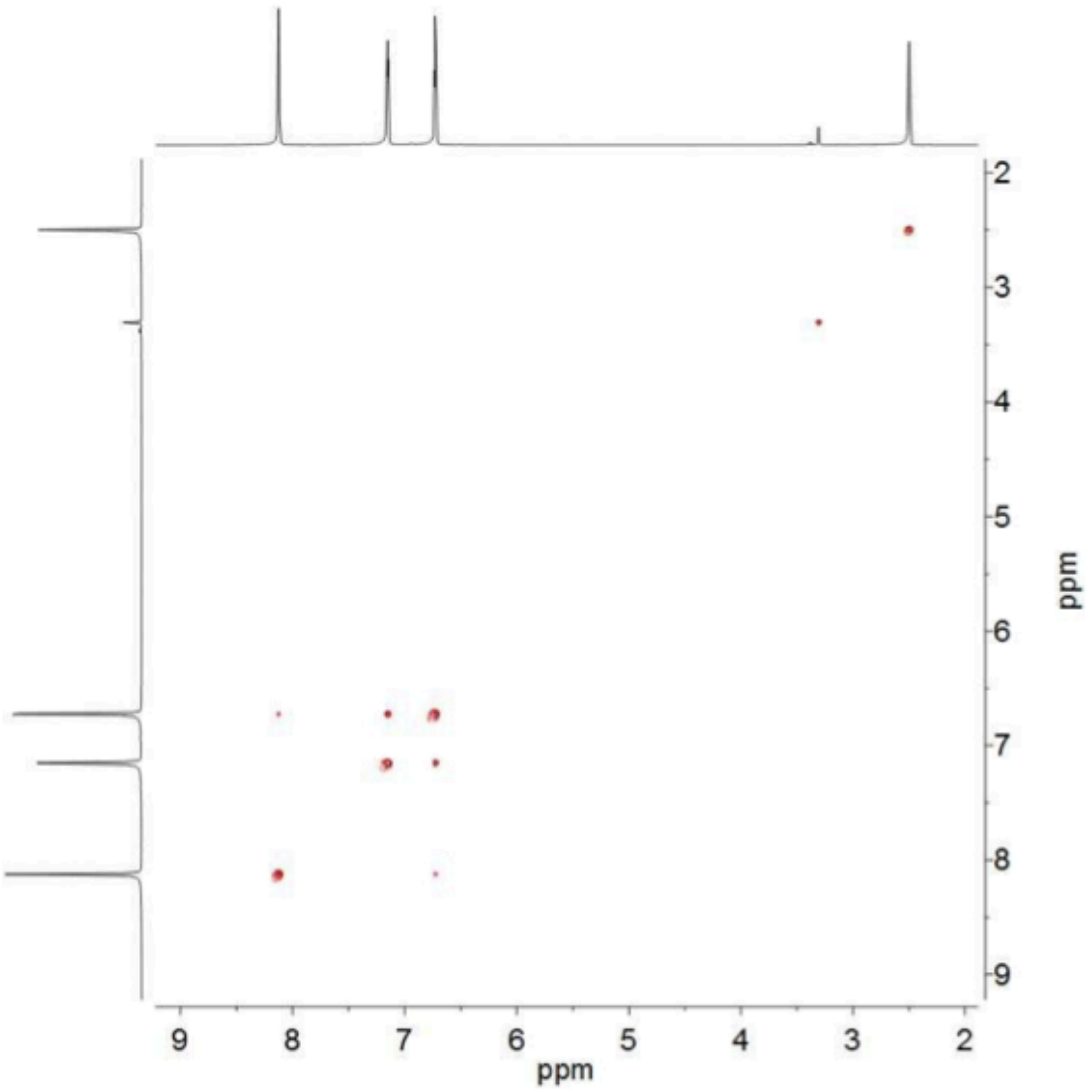


**Supplementary Figure 23.** $^{1}H$ COSY NMR spectrum (DMSO-d6, r.t.) of tri(2-furyl)phosphine oxide.

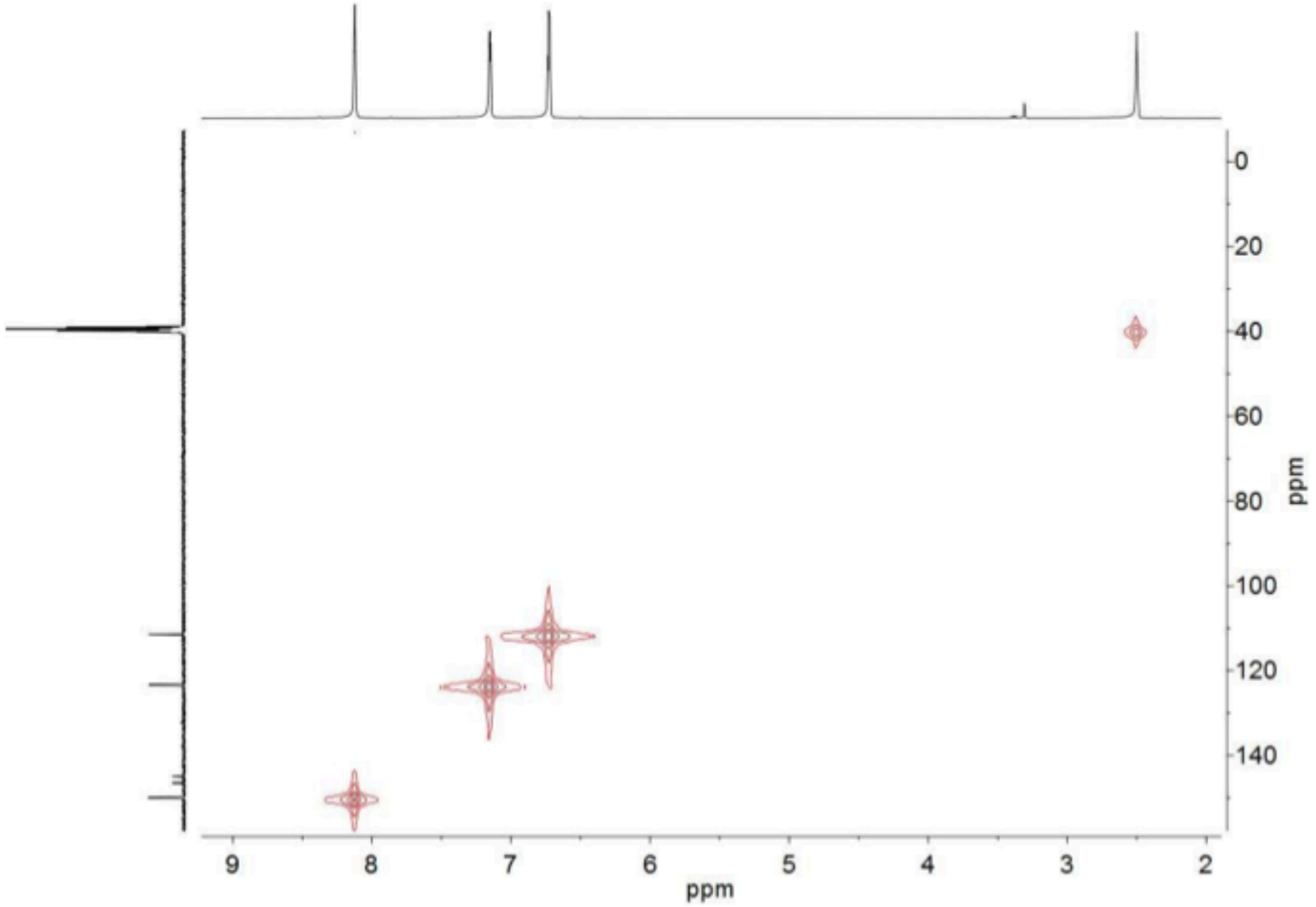


**Supplementary Figure 24.** $^{1}H$-$^{13}C$ HMQC NMR spectrum (DMSO-d6, r.t.) of tri(2-furyl)phosphine oxide

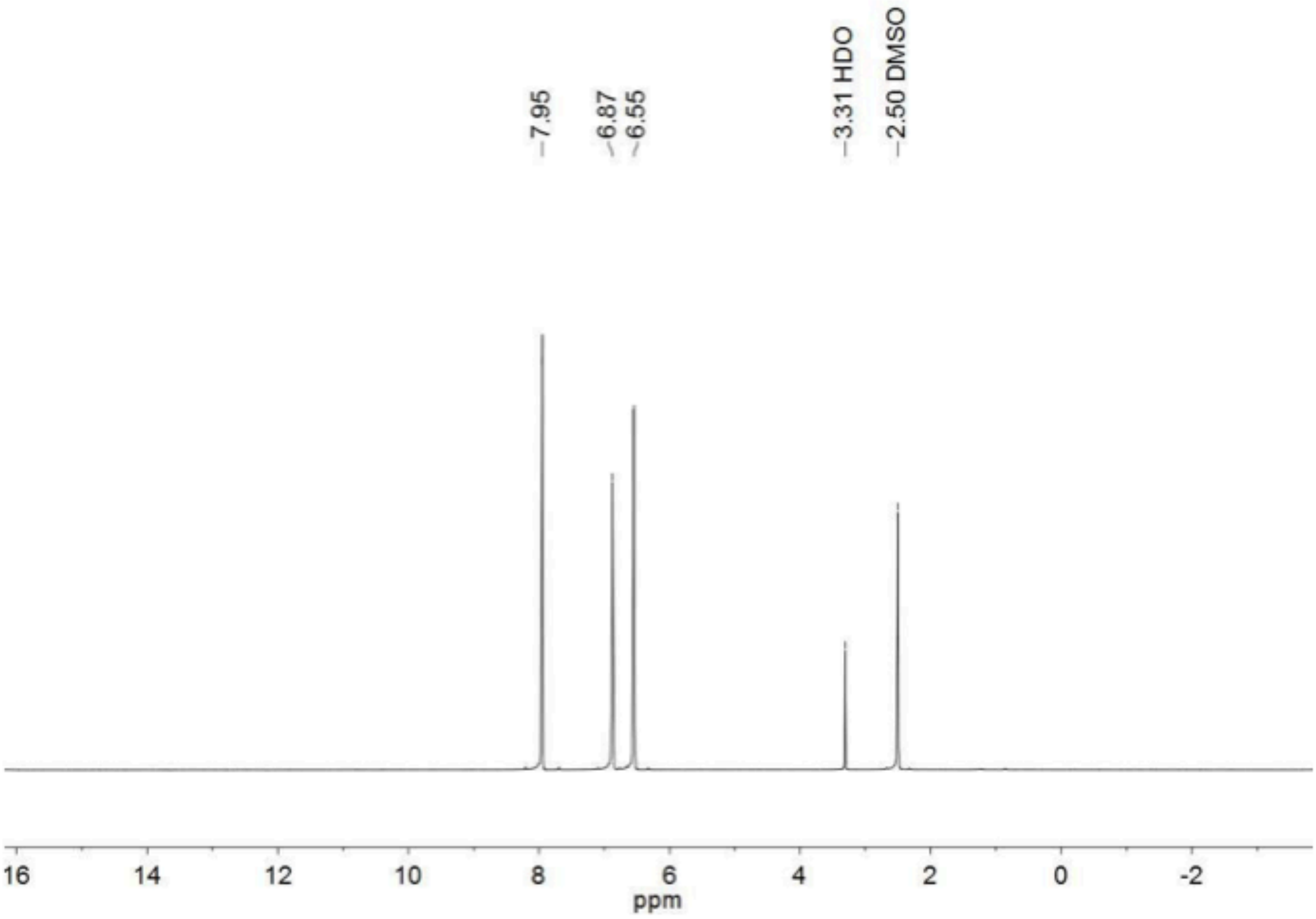


**Supplementary Figure 25.** $^{1}H$ NMR spectrum (DMSO-d6, r.t.) of tri(2-furyl)phosphine.

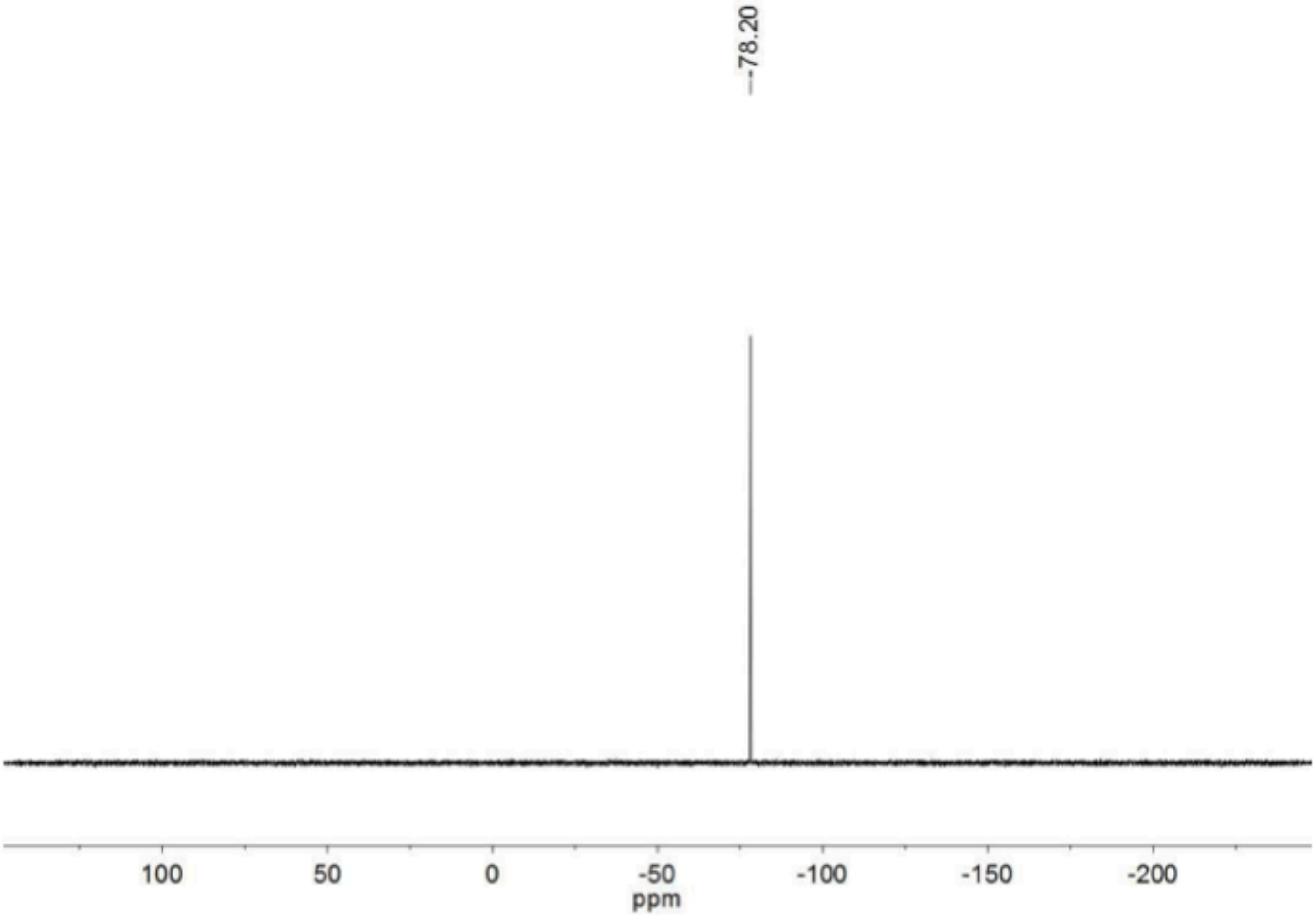


**Supplementary Figure 26.** $^{31}P$ NMR spectrum (DMSO-d6, r.t.) of tri(2-furyl)phosphine.

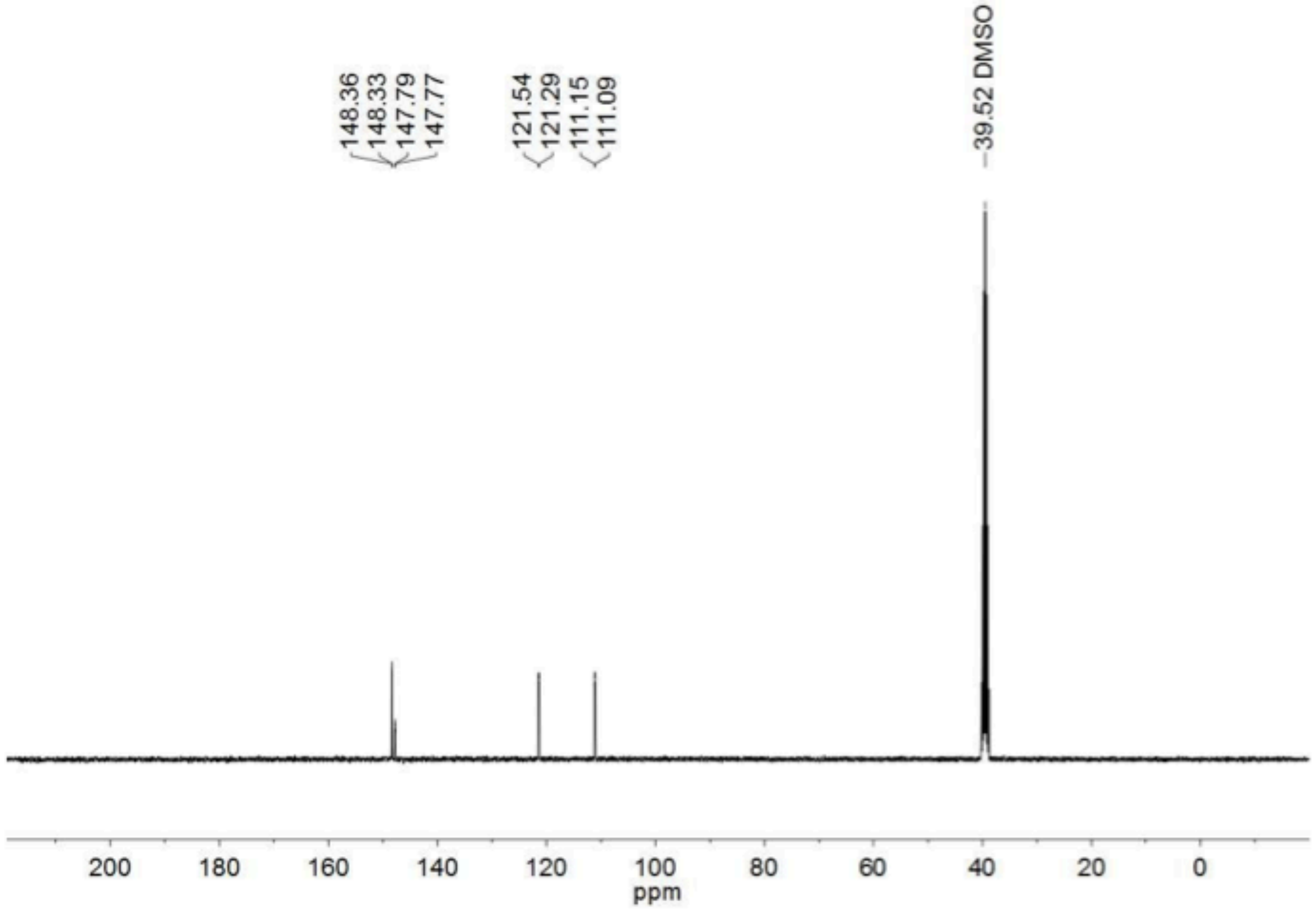


**Supplementary Figure 27.** $^{13}C$ NMR spectrum (DMSO-d6, r.t.) of tri(2-furyl)phosphine.

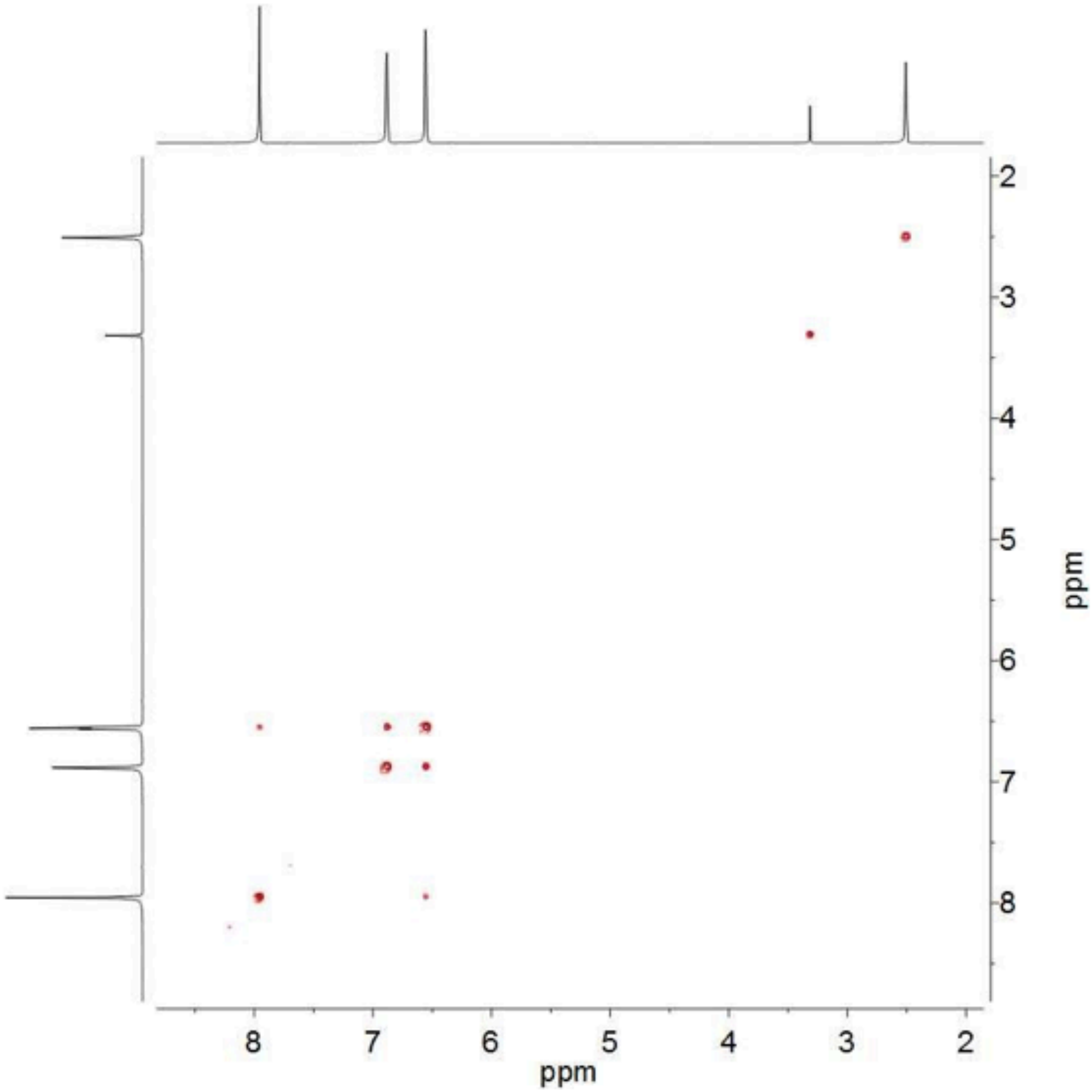


**Supplementary Figure 28.** $^{1}H$ COSY NMR spectrum (DMSO-d6, r.t.) of tri(2-furyl)phosphine.

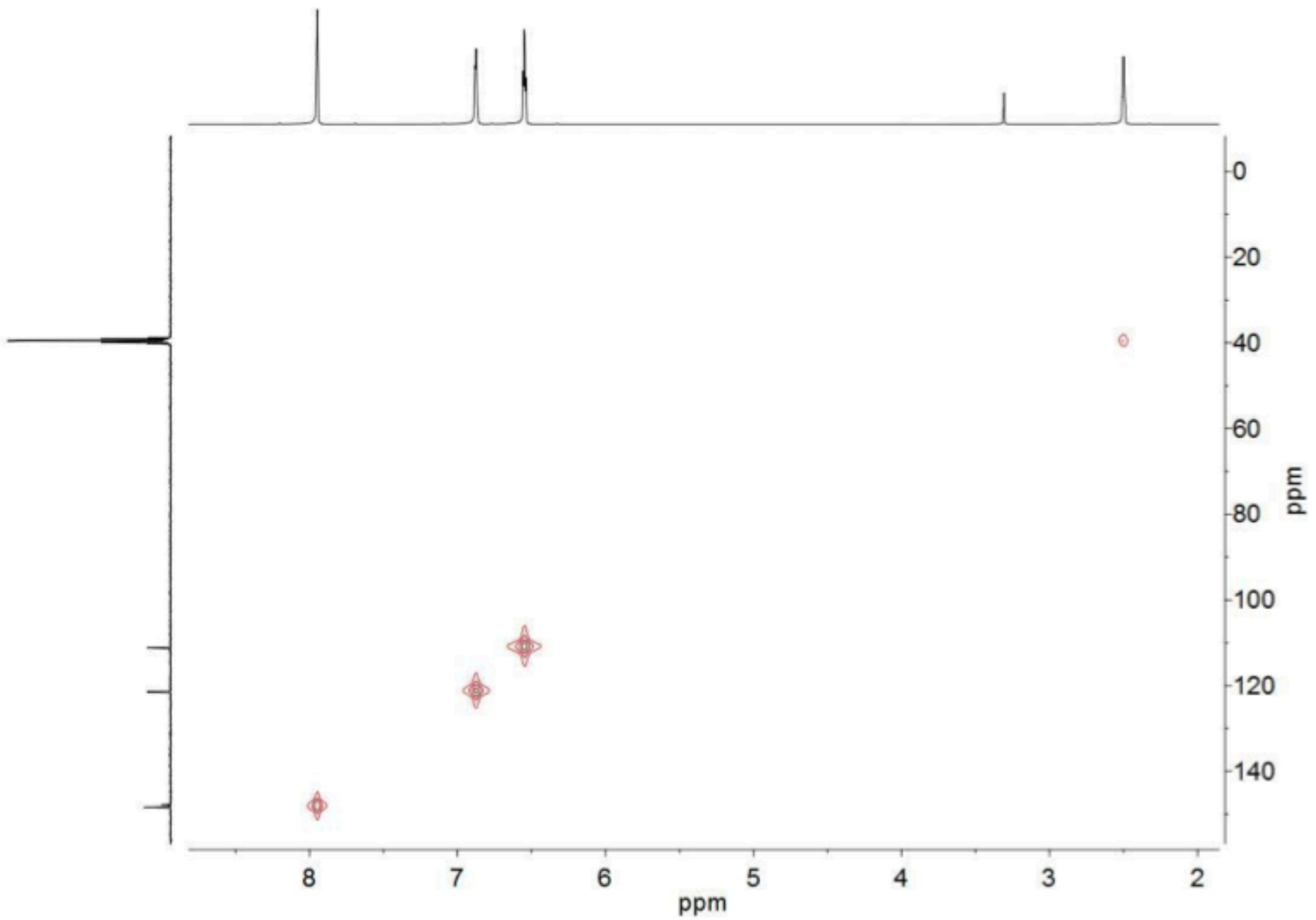


**Supplementary Figure 29.** $^{1}$H-$^{13}$C HMQC NMR spectrum (DMSO-d6, r.t.) of tri(2-furyl)phosphine.

| blood chemistry (n=4) | CTRL | Pd-NP | p-value |
|---|---|---|---|
| BUN (mg/dl) | 30.2 | 26.9 | 0.34 |
| Creatinine (mg/dl) | 0.4 | 0.3 | 0.21 |
| Phosphorus (mg/dl) | 9.5 | 10.0 | 0.76 |
| Calcium (mg/dl) | 9.3 | 8.1 | 0.38 |
| Total Protein (g/dl) | 4.5 | 4.0 | 0.09 |
| Albumin (g/dl) | 2.1 | 1.8 | 0.12 |
| Globulin (g/dl) | 2.4 | 2.3 | 0.07 |
| Glucose (mg/dl) | 215.8 | 178.0 | 0.26 |
| Cholesterol (mg/dl) | 59.8 | 53.3 | 0.22 |
| ALT (GPT) (U/l) | 38.3 | 50.8 | 0.58 |
| ALP (U/l) | 52.5 | 38.00 | 0.003 |
| GGT (U/l) | <10 | <10 | n/a |
| Total Bilirubin (mg/dl) | 1.1 | 0.6 | 0.40 |
| IgE (ug/ml) | 1.1 | 0.7 | 0.19 |

**Supplementary Table 1. Pd-NP impact on clinical blood chemistry.** Measurements were made following 50 mg $kg^{-1}$ treatment with Pd-NP or PLGA-PEG NP vehicle control (q4dx4) 24 h after last treatment in naive BALB/c mice (data are means, n=4), showing p-value according to two-tailed t-test.

| CBC (n=3) | CTRL | Pd-NP | p-value |
|---|---|---|---|
| WBC (1000/ul) | 2.4 | 1.6 | 0.27 |
| LYM (1000/ul) | 1.3 | 0.9 | 0.25 |
| MONO (1000/ul) | 0.3 | 0.2 | 0.29 |
| GRAN (1000/ul) | 0.9 | 0.6 | 0.48 |
| LYM (%) | 56.8 | 55.0 | 0.87 |
| MONO (%) | 9.7 | 10.3 | 0.84 |
| GRAN (%) | 33.5 | 34.7 | 0.89 |
| HCT (%) | 32.8 | 29.2 | 0.34 |
| MCV (fl) | 52.8 | 52.9 | 0.92 |
| RDWa (fl) | 35.4 | 34.1 | 0.07 |
| RDW (%) | 15.7 | 15.0 | 0.28 |
| HGB (g/dl) | 9.9 | 9.0 | 0.36 |
| MCHC (g/dl) | 30.3 | 30.9 | 0.12 |
| MCH (pg) | 16.0 | 16.3 | 0.45 |
| RBC ($10^6$/ul) | 6.2 | 5.5 | 0.32 |
| PLT (1000/ul) | 329.0 | 375.7 | 0.71 |
| MPV (fl) | 6.1 | 7.0 | 0.12 |
| RETIC (%) | 0.8 | 1.0 | 0.43 |

**Supplementary Table 2. Pd-NP impact on complete blood counts.** Complete blood counts were measured in HT1080 tumor-bearing mice after 50 mg $kg^{-1}$ treatment with Pd-NP or PLGA-PEG NP vehicle control (q4dx2) 24 h after last treatment (data are means, n=3, p-value according to two-tailed t-test).

**Supplementary Methods:**

All reagents were purchased from Sigma-Aldrich and used without further purification unless otherwise noted. Deuterated solvents were obtained from Cambridge Isotope Laboratories, Inc. NMR spectra were recorded on a Bruker Avance III 400 spectrometer and calibrated to the residual proton resonance and the natural abundance $^{13}C$ resonance of the solvent (DMSO-d6: d H = 2.50 and d C = 39.52 ppm). Signal multiplicities are abbreviated as: s (singlet), d (doublet), t (triplet), q (quartet), quint (quintet), m (multiplet), br (broad). Silica Gel 60 (40-63 µm) was used for purification. High performance liquid chromatography-mass spectrometry analysis (HPLC/MS) was performed on a Waters instrument equipped with a Waters 2424 ELS Detector, Waters 2998 UV-Vis Diode array Detector, Waters 2475 Multi-wavelength Fluorescence Detector, and a Waters 3100 Mass Detector. Separations employed Waters XTerra RP C18 5 µm, with a water/acetonitrile solvent gradient (0.1% formic acid added). All fluorescence measurements were conducted with a Tecan Safire 2 fluorescence plate reader.

Palladium catalysts were initially selected based on previous publications describing their synthesis and activity under relatively mild reaction conditions[1–11]. $PdCl_2(TPPTS)_2$, $Pd(TPPTS)_4$ and $Pd(TFP)_4$ were synthesized according to published procedures[12,13]. Bis-allyloxycarbonyl- and bis-propargyloxycarbonyl-protected rhodamine 110, and coumarin precursor were all synthesized according to published procedures[14–17]. All catalysts were characterized by NMR for purity and successful synthesis following previously described protocols. In all cases purity was found to be >99%. For the most important compound **1,** prepared as described by Hettrick and Scott[18], NMR, ESI-MS, and elemental analysis are included below. Stability and purity of all catalysts were monitored by NMR throughout the entire study to avoid and eliminate any effects caused by degradation products or impurities.

PLGA(75:25 lactide:glycolide)$_{8.3kDa}$-PEG$_{5.5kDa}$ was purchased from Advanced Polymer Materials, Inc. (Montreal, QC, Canada; Cat. #13-01-CX-75/25-8.3k/5.5k; lot 01-13-146-1). From the vender, gel permeation chromatography (GPC) indicated $M_w$ = 13.8 kDa and PI = 1.38. PEG weight fraction (66%) and MW were confirmed by $^1H$ NMR peak integration values, which were also used to calculate the lactide:glycolide ratio. As described previously[19], BODIPY-630-$NH_2$ (Life Technologies; 2.8 mg, 0.0046 mmol) was conjugated to PLGA(50:50 lactide:glycolide)$_{30\text{-}60kDa}$ (100mg, ca. 0.0023 mmol; Sigma) by stirring under Ar at r.t. for 10 min and adding EDCI (4.4 mg, 0.023 mmol) and DMAP (2.8 mg, 0.023 mmol) in $CH_2Cl_2$ (0.5 mL). After overnight stirring, polymer was precipitated in cold 1:1 MeOH:$Et_2O$, centrifuged (2,700xg, 10 min) and repeatedly washed in minimal $CH_2Cl_2$, followed by MeOH:$Et_2O$ precipitation and centrifugation. The resulting blue precipitate was dried under vacuum.

*Preparation of **1**:* Similar to as described previously[1,18], bis(acetonitrile)dichloropalladium(II) (100 mg, 0.39 mmol, 1.0 eq) was dissolved in dry MeCN (15 mL) and a solution of TFP (178.9 mg, 0.77 mmol, 2.0 eq) in MeCN (2 mL) added. Bright yellow **1** precipitates upon stirring overnight. The crude product is collected and washed twice with water (2 x 10 mL), dissolved in dry methanol and stored at -30$^{o}$C affording **1** as a micro-cystaline powder (140.8 mg, 57%). NMR (DMSO-$d_6$, r.t., [ppm]) $^{1}$H NMR (400 MHz): δ = 8.14-8.05 (s (br), 0.3 H), 7.97-7.89 (m, 3H), 7.24-7.12 (s (br), 0.3 H), 6.98-6.88 (m, 3H), 6.74-6.64 (s (br), 0.3 H), 6.62-6.54 (m, 3H). $^{13}$C NMR (101 MHz): δ = 149.9 (d, JP-C = 5.3 Hz), 139.9 (d, JP-C = 92.4 Hz), 124.8 (d, JP-C = 20.1 Hz), 111.7 (d, JP-C = 7.8 Hz). Intensity 0.3 H and 3H peaks correspond to trans and cis isomers, respectively, in agreement with ref. [18]. $^{31}$P NMR (162 MHz): δ = -22.0 (*cis,* >90%, ref. [18]), -29.0 (*trans,* <10%). Anal. Calcd for $C_{24}H_{18}Cl_2O_6P_2Pd$: C, 44.92; H, 2.83. Found #1: C, 45.15; H, 2.95. Found #2: C, 45.03; H, 2.88 (average results differ from calculated by <2%). LC-MS (ESI) calc for $C_{24}H_{18}Cl_1O_6P_2Pd$ [M-Cl]+ 604.93, found 605.0 (in agreement with ref. [18]). Of note, predominant cis-stereochemistry about a square planar Pd conformation was previously confirmed by crystal structure[18].

*Preparation of tri(2-furyl)phosphine oxide:* Tri(2-furyl)phosphine oxide was synthesized from tri(2-furylphosphine) as previously described[20]. NMR (DMSO-d6, r.t., [ppm]) $^{1}$H NMR (400 MHz): δ = 8.13-8.12 (m, 3H), 7.20-7.12 (m, 3H), 6.75-6.72 (m, 3H). $^{13}$C NMR (101 MHz): δ = 149.9 (d, JP-C = 8.3 Hz), 145.7 (d, JP-C = 156.2 Hz), 123.4 (d, JP-C = 22.0 Hz), 111.4 (d, JP-C = 9.3 Hz). $^{31}$P NMR (162 MHz): δ = -13.7.

*NMR data of tri(2-furyl)phosphine:* NMR (DMSO-d6, r.t., [ppm]) $^{1}$H NMR (400 MHz): δ = 7.95 (d, J = 1.7 Hz, 3H), 6.90-6.87 (m, 3H), 6.57-6.54 (m, 3H). $^{13}$C NMR (101 MHz): δ = 148.3 (d, JP-C = 2.9 Hz), 147.8 (d, J = 2.4 Hz), 121.4 (d, JP-C = 24.9 Hz), 111.1 (d, JP-C = 6.3 Hz). $^{31}$P NMR (162 MHz) δ = -78.2.

*Preparation of alloc-doxorubicin (proDOX):* Following a previously described procedure [21] we observed formation of bis-allyloxycarbonyl-substituted doxorubicin as an inseparable by-product of proDOX. For that reason we modified this procedure as follows: doxorubicin hydrochloride (580 mg, 1.0 mmol) was suspended in anhydrous DMF (100 mL) and 4-(dimethylamino)pyridine (366.5 mg, 3.0 mmol, 3 eq) was added. After stirring at room temperature for 15 min the solution was cooled to -78°C and allyl chloroformate (106.3 $\mu$L, 1.0 mmol, 1 eq) dissolved in anhydrous DMF (20 mL) was dropwise added within 10 min. The mixture was slowly warmed to room temperature and stirring was continued for 5 h. HPLC analysis showed ~50% conversion, which is why the solution was again cooled to -78°C followed by addition of further allyl chloroformate (53.2 $\mu$L, 0.5 mmol, 0.5 eq) in anhydrous DMF (10 mL). The mixture was slowly warmed to room temperature and stirring was

continued for 2 h. The solvent was evaporated and the residue was purified by reverse phase chromatography (60 g $C_{18}$ silica, water/acetonitrile gradient elution, 0.1% formic acid added) to obtain pure proDOX as a red solid (490 mg, 78%). NMR data matched that reported previously[21]. LC-MS (ESI) calc for $C_{31}H_{32}NO_{13}$ [M-H]- 626.2, found 626.06 (Supplementary Fig. 11a).

**In vitro NP characterization:** For cytotoxicity assays, 5,000 cells per well were added to 96-well plates, treated the next day with compound or the appropriate buffer control (DMF or drug-free PLGA-PEG nanoparticles, as appropriate), and assessed for viability 72 hr later using PrestoBlue (Life Technologies) following the manufacturer's protocol. $IC_{50}$ values for each compound were calculated by interpolating from an 11-point dose-response curve.

For live-cell *in vitro* microscopy of Pd-NP uptake and catalytic activity, HT1080-53BP1-mApple cells were seeded 20,000 cells per well on an optical-bottom 96-well plate (Ibidi; Applied Biophysics), treated the following day with indicated concentrations of Pd-NP, $Alloc_2R110$ NP, or coumarin precursor for 24 hr, washed in warm medium, and immediately imaged on a DeltaVision (Applied Precision) modified Olympus BX63 microscopy system with an environmental chamber using the following excitation / emission wavelengths (in nm): 360/455 (Pd-NP), 490/525 (R110), 360/525-605 (coumarin), and 555/605 (53BP1-mApple). In vitro measurement of coumarin fluorescence kinetics were performed in PBS at 37°C on a fluorescence plate reader (TECAN), with $\lambda_{ex}$ / $\lambda_{em}$ = 375/500. For solubilization, all experiments with coumarin precursor involved first adding 0.1% vol/vol coumarin precursor (an oil) to 10% bovine serum albumin (BSA; Sigma) in PBS; sonicating and vortexing at 37°C for 30 min, and adding to cells or Pd-NP solutions at the indicated concentration. Coumarin-free BSA solution was used as the vehicle control where appropriate.

Measurements of intracellular Pd uptake using ICP-MS were performed by treating 80% confluent cells in 20 cm plates for 24 hr with Pd-NP or un-encapsulated $PdCl_2(TFP)_2$, rinsing 3x with PBS, trypsinizing, centrifuging the cell pellet for 300xg for 5 min, re-suspending the pellet in lysis buffer (150 mM NaCl; 1% Triton X-100; 50 mM Tris pH 8.0), and freezing it for ICP-MS analysis (Galbraith Labs).

For *in vitro* NP payload release measurements, samples were incubated in PBS at 37°C and filtered using 30 kDa molecular weight cutoff filters (Millipore Amicon) at the indicated time-points. Flow-through was measured for drug content by absorbance using a Nanodrop spectrophotometer. At the end of the time-course, NP was dissolved in DMF and measured for drug content by absorbance. Additionally, flow-through and remaining NP was assayed for activity by incubation with 10 µg $mL^{-1}$ $Alloc_2R110$ in PBS + 10% DMF for 2 h. Initial

concentrations were 500 µM. For tested release across multiple buffers (Supplementary Fig. 2f), samples were incubated at 37$^{o}$C and filtered using 100 kDa molecular weight cutoff filters (Millipore Amicon) at a single time-point, with an initial concentration of 1mM (12 h). Flow-through was measured for drug content by absorbance using a Nanodrop spectrophotometer, after accounting for buffer-dependent absorbance properties. DMEM + 10% FBS + L-glutamine and Pen/Strep was used as the growth media. Lysis buffer consisted of 150 mM NaCl, 0.1% Triton X-100, 50 mM Tris-HCl, pH 8.0. In the figure, T-X denotes Triton X-100.

**Computational image analysis:** Unless otherwise stated, all experiments were independently performed at least twice as biological replicates, and t-tests assumed normal or log-normal distribution of data with equal variance. Where appropriate, equal variance was confirmed by F-test and normality was confirmed by the D'Agostino and Pearson omnibus test (Prism GraphPad); when tests showed non-Gaussian distribution, non-parametric Mann-Whitney U test was used (Fig. 5c-d). Intravital microscopy images were analyzed using either Matlab (Mathworks) or ImageJ and were pre-processed using background subtraction based on data acquired immediately prior to NP injection. For vascular half-life calculations (Fig. 3), fluorescence time-lapse values from multiple vessels across multiple animals were recorded, averaged, and fit to an exponential decay model using nonlinear regression in MATLAB. From *in vitro s*ingle-cell fluorescence intensities were determined through automated segmentation and quantification algorithms using CellProfiler[22]. Fluorescence turn-on was calculated by the ratio of mean fluorescence [Pd+substrate] / [substrate] after subtracting background fluorescence measured in the untreated control. Max in vivo turn-on (Supplementary Fig. 8) was calculating using the ratio of fluorescence, but using the fluorescence quantile yielding max turn-on (97$^{th}$ percentile) rather than the simple mean. Average 53BP1 puncta (Fig. 5b) was determined by counting foci per individual cell, averaging values across the entire cell population (n > 100), and normalizing these values to the untreated control values. Nuclear drug accumulation (Fig. 6c) was determined by integrating fluorescence intensity that co-localized with Hoechst DNA counterstain compared to elsewhere in the cell, averaged across a population of single-cells across multiple (n = 4) tumors.

**Nuclear doxorubicin imaging:** 24 h following drug treatment, mice bearing intrabursal A2780CP ovarian cancer tumors were treated with lectin-DyLight649 Lycopersicon Esculentum (Tomato) Lectin (Vector Labs) for 30 min to label vasculature, anesthetized, perfused with formalin via cardiac puncture, and organs were removed for 6 h formalin fixation at r.t. To reduce autofluorescence, tissue was then cleared under rocking agitation at 37$^{o}$C for 12 h according to previously published methods[23] in the presence of 0.1% Hoechst 33342, and immediately imaged with an Olympus FV1000 confocal-multiphoton imaging system. ProDOX was dosed

at 32 μmol $kg^{-1}$ while DOX and DOX-NP was dosed at the MTD of 16 μmol $kg^{-1}$. For Supplementary Fig. 15a, lectin-Rhodamine Griffonia Simplicifolia Lectin I (Vector Labs) was used. 16 μmol $kg^{-1}$ proDOX encapsulated in PLGA-BODIPY630 labeled PLGA-PEG NP was used in Supplementary Fig. 15b-d, and organs were imaged immediately following sacrifice and excision, without fixation.

**γH2A.X analysis:** For quantification of DNA content and DNA damage response (Fig. 4d), cells were plated O/N at 80% confluency in 10 cm plates, treated for 24 h, rinsed, trypsinized, and treated with LIVE/DEAD fixable violet dead cell stain (Life Technologies) to exclude dead cells. Cells were then fixed in 70% ethanol overnight at -20°C, rinsed, permeabilized in 0.1% Triton-X-100, blocked for 1 h in Odyssey Blocking Buffer (Li-Cor), and stained for γH2A.X (clone 20E3; Cell Signaling Technology) in conjunction with FxCycle Far Red DNA counterstain (Life Technologies). Cells were treated with 100 μg $mL^{-1}$ RNaseA (Thermo) and rinsed before analysis. G1/S/G2M populations were manually gated in Matlab based on DNA content. For γH2A.X immunofluorescence microscopy (Fig. 4f), cells were plated O/N at 80% confluency in 96-well optical bottom plates (Ibidi), treated for 24 h, rinsed, fixed for 15 min with 4% paraformaldehyde at r.t., permeabilized in PBS + 0.1% Triton-X-100 for 30 min, blocked for 1 h using Odyssey Blocking Buffer (Li-Cor), applied γH2A.X (clone 20E3; Cell Signaling Technology) in blocking buffer O/N, rinsed, and imaged using a modified Olympus BX63 microscopy system.

**Histology and flow cytometry:** Subcutaneous HT1080 tumors were harvested 3 weeks post-implantation and 24 h post-treatment with PLGA-PEG therapeutic nanoparticles[24] co-encapsulated with PLGA-BODIPY630. Flow cytometry and histology are both described previously[24]. For histology, excised tumors were embedded in O.C.T. compound (Sakura Finetek). Fresh-frozen 6 μm thick sections were prepared and stained with anti-mouse F4/80 antibody (clone: BM8, eBioscience), anti-rat IgG-Alexa Fluor 405 (Abcam) as a secondary antibody, and the images were captured using BX63 (Olympus) equipped with ANDOR Neo sCMOS Monochrome Camera (Andor Technology Ltd.). For flow cytometry analysis, HT1080 tumors were harvested from nu/nu mice three weeks after subcutaneous implantation and processed as described previously[24]. The following cell types were identified by cell marker expression: tumor cells ($hCD29^{+}$), macrophages ($CD45^{+}CD11b^{+}Ly6C^{-}Lin^{-}CD11c^{+}F4/80^{+}$), neutrophils ($CD45^{+}CD11b^{+}Ly6C^{+}Lin^{+}$) and lymphocyte-like cells ($CD45^{+}Lin^{+}CD11b^{-}Ly6G^{-}$). The lineage mixture (Lin) included anti-CD90.2, anti-B220, anti-NK1.1, anti-CD49b, anti-Ter119 and anti-Ly6G. NIR-labeled NP (PLGA-BODIPY-630) fluorescence was assessed by using an LSRII flow cytometer and the mean fluorescence intensity (MFI) of the different cell populations was determined. Background autofluorescence for each immunologically defined cell population was measured from control-treated animals that were injected with non-fluorescent PLGA-PEG vehicle; this value was

subtracted from the data for the fluorescent TNP-treated animals.

**Bioluminescence imaging**: To assess tumor burden in the A2780CP and ID8 orthotopic tumor models, bioluminescence imaging was performed by stable transgenic overexpression of firefly luciferase [25], and imaging was performed using the SPECTRAL Ami-X (Spectral Instruments Imaging). Animals were anesthetized using isoflurane, injected with 7 mg D-Luciferin i.p., imaged every 5 minutes until maximum radiance was achieved, and concordantly x-ray imaged for anatomical reference.

**Biodistribution:** Palladium uptake into tissues was assessed by ICP-MS. Briefly, organs were harvested 24 h post-treatment after blood draw via cardiac puncture and cardiac perfusion with 10 mL PBS under isoflurane anesthesia. As previously described[26], 10-150 mg tissue was added to 1.5 mL 70% nitric acid, digested at 40$^{o}$C for 24 h, and diluted 1:30 into 3% nitric acid aqueous solution for analysis using an Agilant 7500 Series ICP-MS. Data were fit to a 9-point standard curve ($R^2 > 0.999$; L.O.D. < 0.1 ppb) using Pd ICP standard solution (Millipore). For proDOX and DOX quantification, tissue was excised after cardiac perfusion with 10 mL PBS and free drug was extracted following previously published protocols, including use of 1 µM daunorubicin internal standard [27]. Concentrations were fit from integrated fluorescence chromatography (Supplementary Fig. 12c) and fit to standard dilution series for DOX ($R^2 > 0.96$; L.O.D. = 0.1 µM) and proDOX ($R^2 > 0.99$; L.O.D. = 0.01 µM). In vitro quantification was performed after washing 10cm confluent plates of HT1080 3 times in PBS, lysing using 50 µl lysis buffer (150 mM NaCl, 1% Triton X-100, 50 mM Tris, Roche cOmplete protease inhibitor, pH 8.0), and extracting drug as described[27]. Molar concentrations in tissue assumed density of 1 kg $L^{-1}$ and were estimated as $[\%I.D./g] * [total\ mass\ I.D.] * density_{tissue} * MW^{-1}$.

**Toxicity:** Complete blood count and blood chemistry panels were performed by the MGH Center for Comparative Medicine Veterinary Clinical Pathology Laboratory. Blood draws were performed by cardiac puncture into EDTA coated BD microtainers for blood count (which included manual reticulocyte count), and into lithium-heparin BD microtainers for blood chemistry analysis. Murine IgE was measured according to manufacturer's guidelines (eBioscience Ready-SET-Go ELISA), with reference to a 6-point standard curve ($R^2 > 0.97$).

**Solvent Pd administration:** In order to safely administer un-encapsulated $PdCl_2(TFP)_2$ into tumor-bearing mice, complex was dissolved in DMSO at 40 mg $ml^{-1}$, added to 7 $\mu$l DMAC/Solutol and 100 $\mu$l PBS, vortexed and sonicated, and slowly administered via tail-vein catheter and syringe pump while the animal was under isoflurane anesthesia. DMSO was confirmed to not significantly impact $Alloc_2R110$ activation, compared to

DMF solvent, in an *in vitro* assay in HBSS buffer as in Fig. 1 ($p>0.05$; $n=3$).

**Supplementary References:**


1. Zeng, X., Hu, Q., Qian, M. & Negishi, E. Clean inversion of configuration in the Pd-catalyzed cross-coupling of 2-bromo-1,3-dienes. *J Am Chem Soc* **125**, 13636-13637 (2003).
2. Bouyssi, D., Gerusz, V. & Balme, G. A Novel Palladium Catalyst for Cross Coupling of Allyl Acetates with Arylboronic Acids. *Eur J Org Chem* **2002**, 2445-2448 (2002).
3. Liu, W. et al. Water-soluble colorimetric and ratiometric fluorescent probe for selective imaging of palladium species in living cells. *Inorg Chem* **53**, 12590-12594 (2014).
4. Hosomi, A., Kohra, S. & Tominaga, Y. Pentacoordinate organosilicon compounds in organic synthesis. Cross-coupling of alkenylsiliconates with organic halides and triflates catalyzed by palladium complex. *Chem Pharm Bull* **36**, 4622-4625 (1988).
5. Feuerstein, M., Doucet, H. & Santelli, M. Efficient Heck vinylation of aryl halides catalyzed by a new air-stable palladium-tetraphosphine complex. *J Org Chem* **66**, 5923-5925 (2001).
6. Viciu, M. S. et al. Synthetic and Structural Studies of (NHC) Pd (allyl) Cl Complexes (NHC= N-heterocyclic carbene). *Organometallics* **23**, 1629-1635 (2004).
7. Amatore, C., Broeker, G., Jutand, A. & Khalil, F. Identification of the effective palladium (0) catalytic species generated in situ from mixtures of Pd (dba) 2 and bidentate phosphine ligands. Determination of their rates and mechanism in oxidative addition. *J Am Chem Soc* **119**, 5176-5185 (1997).
8. Kostas, I. D., Andreadaki, F. J., Kovala-Demertzi, D. & Prentjas, C. D., MA. Suzuki–Miyaura cross-coupling reaction of aryl bromides and chlorides with phenylboronic acid under aerobic conditions catalyzed by palladium complexes with thiosemicarbazone ligands. *Tetrahedron Lett* **46**, 1967-1970 (2005).
9. Ito, H., Kusukawa, T. & Fujita, M. Wacker Oxidation in an Aqueous Phase through the Reverse Phase-Transfer Catalysis of a Self-Assembled Nanocage. *Chem Lett* **6**, 598-599
10. Watson, D. A. et al. Formation of ArF from LPdAr(F): catalytic conversion of aryl triflates to aryl fluorides. *Science* **325**, 1661-1664 (2009).
11. Wu, X. F., Anbarasan, P., Neumann, H. & Beller, M. Palladium-catalyzed carbonylative C-H activation of heteroarenes. *Angew Chem Int Ed Engl* **49**, 7316-7319 (2010).
12. Wang, J. et al. Chemical remodeling of cell-surface sialic acids through a palladium-triggered bioorthogonal elimination reaction. *Angew Chem Int Ed Engl* **54**, 5364-5368 (2015).
13. Elliott, E. L., Ray, C. R., Kraft, S., Atkins, J. R. & Moore, J. S. Solid-phase synthesis of m-phenylene ethynylene heterosequence oligomers. *J Org Chem* **71**, 5282-5290 (2006).
14. Völker, T., Dempwolff, F., Graumann, P. L. & Meggers, E. Progress towards bioorthogonal catalysis with organometallic compounds. *Angew Chem Int Ed Engl* **53**, 10536-10540 (2014).
15. Streu, C. & Meggers, E. Ruthenium-induced allylcarbamate cleavage in living cells. *Angew Chem Int Ed Engl* **45**, 5645-5648 (2006).
16. Wu, Q. & Anslyn, E. V. Heavy metal analysis using a Heck-catalyzed cyclization to create coumarin. *J Mater Chem* **15**, 2815-2819 (2005).
17. Li, J. et al. Palladium-triggered deprotection chemistry for protein activation in living cells. *Nat Chem* **6**, 352-361 (2014).
18. Hettrick, C. M. & Scott, W. J. Palladium-catalyzed oxyhexatriene cyclization: a novel approach to cyclohexenone annulation. *J Am Chem Soc* **113**, 4903-4910 (1991).
19. Laughney, A. M. et al. Single-cell pharmacokinetic imaging reveals a therapeutic strategy to overcome drug resistance to the microtubule inhibitor eribulin. *Sci Transl Med* **6**, 261ra152 (2014).
20. Griffin, C. E., Peller, R. P., Martin, K. R. & Peters, J. A. Preparation and ultraviolet spectra of tri-2-heteroarylphosphine oxides. *J Org Chem* **30**, 97-101 (1965).

21. Cotterill, I. C., Rich, J. O., Scholten, M. D., Mozhaeva, L. & Michels, P. C. Reversible derivatization to enhance enzymatic synthesis: chemoenzymatic synthesis of doxorubicin-14-O-esters. *Biotechnol Bioeng* **101**, 435-440 (2008).
22. Carpenter, A. E. et al. CellProfiler: image analysis software for identifying and quantifying cell phenotypes. *Genome Biol* **7**, R100 (2006).
23. Susaki, E. A. et al. Whole-brain imaging with single-cell resolution using chemical cocktails and computational analysis. *Cell* **157**, 726-739 (2014).
24. Miller, M. A. et al. Tumour-associated macrophages act as a slow-release reservoir of nano-therapeutic Pt(IV) pro-drug. *Nat Commun* **6**, 8692 (2015).
25. Naba, A., Clauser, K. R., Lamar, J. M., Carr, S. A. & Hynes, R. O. Extracellular matrix signatures of human mammary carcinoma identify novel metastasis promoters. *Elife* **3**, e01308 (2014).
26. Huang, S. et al. Molecular MRI of acute necrosis with a novel DNA-binding gadolinium chelate: kinetics of cell death and clearance in infarcted myocardium. *Circ Cardiovasc Imaging* **4**, 729-737 (2011).
27. Albright, C. F. et al. Matrix metalloproteinase-activated doxorubicin prodrugs inhibit HT1080 xenograft growth better than doxorubicin with less toxicity. *Mol Cancer Ther* **4**, 751-760 (2005).
